# RbFe(HAsO_4_)_2_ and TlFe(HAsO_4_)_2_, two new hydrogenarsenates adopting two closely related structure types

**DOI:** 10.1107/S2056989018006473

**Published:** 2018-05-01

**Authors:** Karolina Schwendtner, Uwe Kolitsch

**Affiliations:** a Institute for Chemical Technology and Analytics, Division of Structural Chemistry, TU Wien, Getreidemarkt 9/164-SC, 1060 Vienna, Austria; b Naturhistorisches Museum, Burgring 7, 1010 Wien, and Institut für Mineralogie und Kristallographie, Universität Wien, Althanstrasse 14, 1090 Wien, Austria

**Keywords:** crystal structure, RbFe(HAsO_4_)_2_, TlFe(HAsO_4_)_2_, arsenate, hydrogenarsenate(V), framework structure, cation disorder

## Abstract

Rubidium iron bis­[hydrogen arsenate(V)], RbFe(HAsO_4_)_2_, and thallium iron bis­[hydrogen arsenate(V)], TlFe(HAsO_4_)_2_, were grown under mild hydro­thermal conditions (*T* = 493 K, 7 d). RbFe(HAsO_4_)_2_ adopts the RbFe(HPO_4_)_2_ structure type (space group *R*  *c*), while TlFe(HAsO_4_)_2_ crystallizes in the (NH_4_)Fe(HPO_4_)_2_ structure type (space group *P* ). Both compounds have tetra­hedral–octa­hedral framework topologies. The *M* ^+^ cations are located in channels of the respective framework and are disordered in TlFe(HAsO_4_)_2_, which may suggest that the *M* ^+^ cations can move in the channels.

## Abstract 2

Rubidium iron bis­[hydrogen arsenate(V)] and thallium iron bis­[hydrogen arsenate(V)] were grown under mild hydro­thermal conditions (*T* = 493 K, 7 d). RbFe(HAsO_4_)_2_ adopts the RbFe(HPO_4_)_2_ structure type (space group *R* 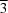 *c*), while TlFe(HAsO_4_)_2_ crystallizes in the (NH_4_)Fe(HPO_4_)_2_ structure type (space group *P* 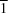).

## Chemical context   

Compounds with mixed tetra­hedral–octa­hedral (T–O) framework structures exhibit a broad range of different topologies, resulting in structures with various inter­esting properties. Arsenates, similar to phosphates or silicates, tend to form T–O framework structures, with properties such as ion conductivity (Chouchene *et al.*, 2017 ▸; d’Yvoire *et al.*, 1983 ▸, 1986 ▸, 1988 ▸; Masquelier *et al.*, 1990 ▸, 1994 ▸, 1995 ▸, 1996 ▸,1998 ▸; Ouerfelli *et al.*, 2007*a* ▸, 2008 ▸; Pintard-Scrépel *et al.*, 1983 ▸) and ion exchange (Masquelier *et al.*, 1996 ▸), as well as unusual piezoelectric (Cambon *et al.*, 2003 ▸, 2005 ▸; Krempl, 2005 ▸; Ren *et al.*, 2015 ▸), magnetic (Ouerfelli *et al.*, 2007*b* ▸) or non-linear optical features (frequency doubling) (Carvajal *et al.*, 2005 ▸; Kato, 1975 ▸; Sun *et al.*, 2017 ▸). To further increase the know­ledge about the possible compounds and structure types of arsenates, a comprehensive study of the system *M* ^+^–*M* ^3+^–O–(H)–As^5+^ (*M* ^+^ = Li, Na, K, Rb, Cs, Ag, Tl, NH_4_; *M* ^3+^ = Al, Ga, In, Sc, Fe, Cr, Tl) was undertaken, which led to a large number of new compounds, most of which have been published (Schwendtner & Kolitsch, 2004 ▸, 2017 ▸, 2018 ▸ and references therein).

**Figure 1:**
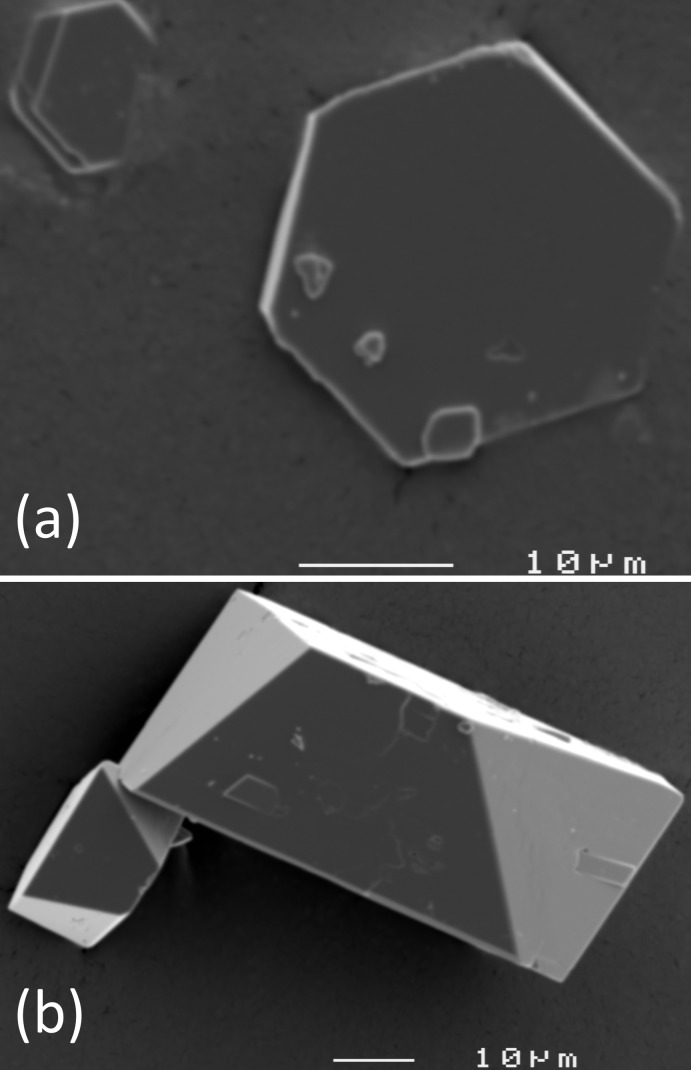
SEM micrographs of crystals of (*a*) RbFe(HAsO_4_)_2_ and (*b*) TlFe(HAsO_4_)_2_.

Among the many different structure types found during our study, one atomic arrangement, the RbFe(HPO_4_)_2_ type (Lii & Wu, 1994 ▸; rhombohedral, *R* 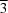 *c*), was found to be extremely versatile, allowing the incorporation of a wide variety of cations. Representatives of this structure type are presently known among arsenates and phosphates containing Rb or Cs as the *M* ^+^ cation and Al, Ga, Fe, In as *M* ^3+^; see Table 1 ▸ for a complete compilation of these compounds. RbFe(HAsO_4_)_2_ (Fig. 1 ▸ *a*) is the fifth arsenate adopting this structure type. There is only one other Rb–Fe–arsenate known to date, Rb_2_Fe_2_O(AsO_4_)_2_ (Chang *et al.*, 1997 ▸; Garlea *et al.*, 2014 ▸). The literature reports one arsenate containing Tl and Fe, the diarsenate TlFe_0.22_Al_0.78_As_2_O_7_ (Ouerfelli *et al.*, 2007*a* ▸); however, the second title compound, TlFe(HAsO_4_)_2_ (Fig. 1 ▸ *b*), is the sole arsenate containing only Tl and Fe to date. It adopts the triclinic (*P* 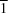) (NH_4_)Fe(HPO_4_)_2_ structure type (Yakubovich, 1993 ▸), along with CsSc(HAsO_4_)_2_ (Schwendtner & Kolitsch, 2004 ▸) and (NH_4_)Fe(HAsO_4_)_2_ (Ouerfelli *et al.*, 2014 ▸) as arsenate members and a wide variety of phosphate members (see compilation in Table 1 ▸). These two structure types are closely related, the (NH_4_)Fe(HPO_4_)_2_ structure type (Yakubovich, 1993 ▸) representing a distorted version of the RbFe(HPO_4_)_2_-type atomic arrangement (Lii & Wu, 1994 ▸).

## Structural commentary   

**Figure 2:**
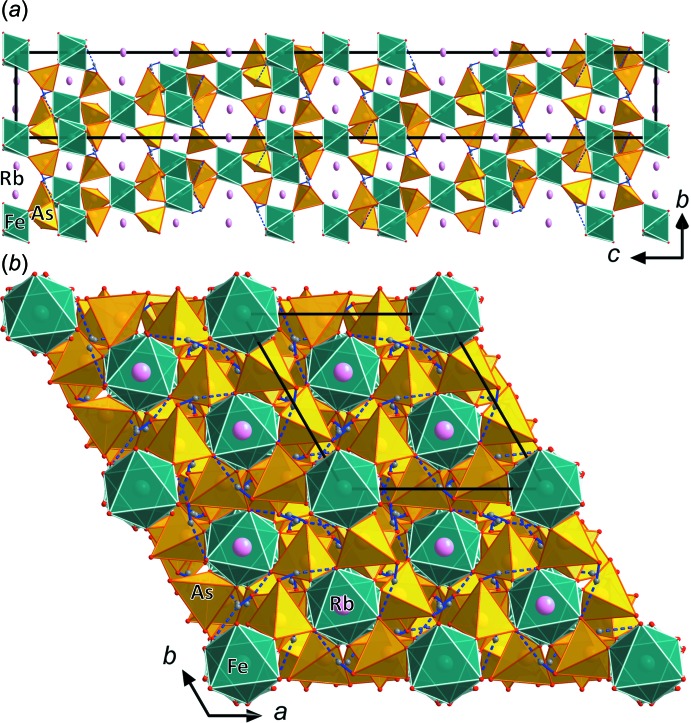
Structure drawing of RbFe(HAsO_4_)_2_ along (*a*) [100] and (*b*) [001]. The Rb atoms, located in channels of the framework structure, are shown with displacement ellipsoids at the 70% probability level. Hydrogen bonds are shown as dashed lines.

**Figure 3:**
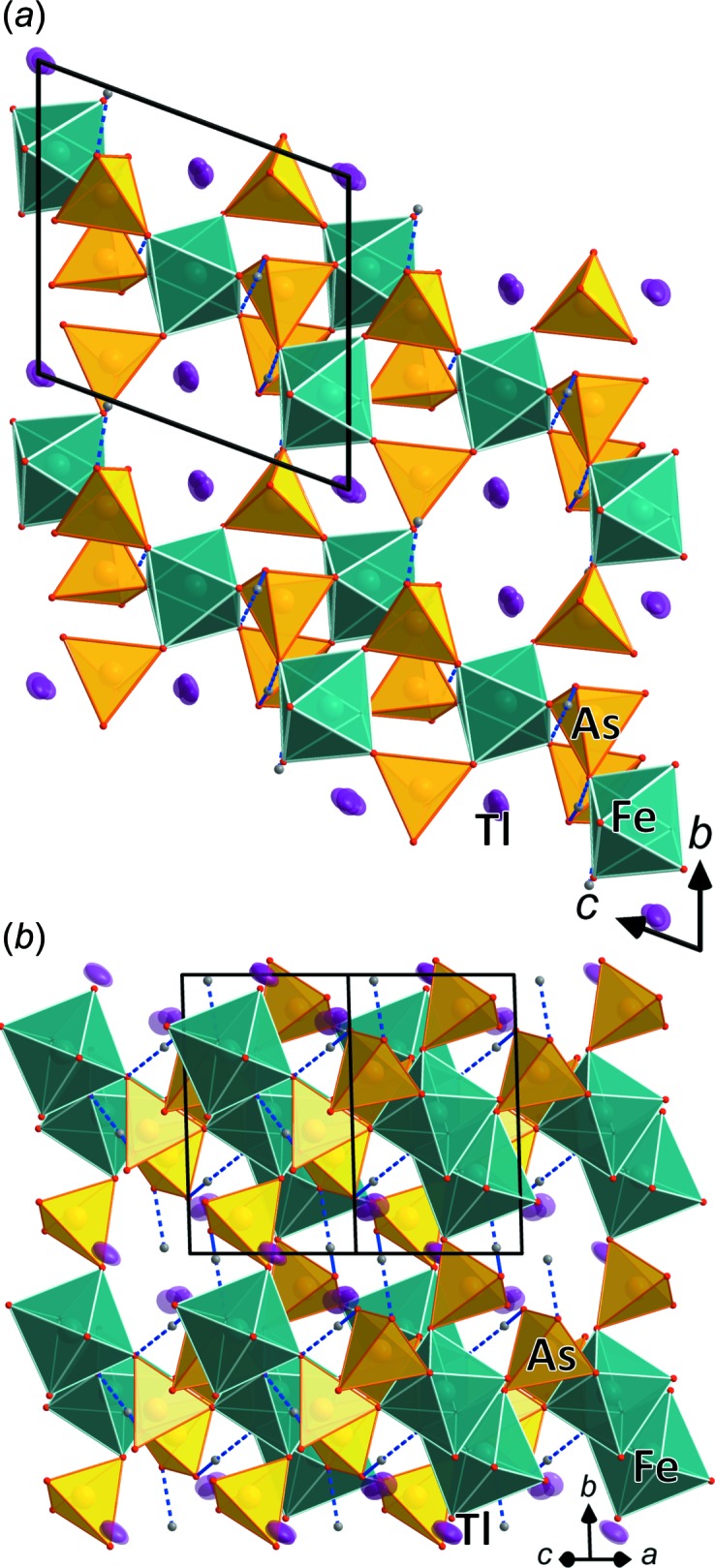
Structure drawing of TlFe(HAsO_4_)_2_ along (*a*) [100] and (*b*) [101]. The disordered Tl atoms are shown with displacement ellipsoids at the 70% probability level. Hydrogen bonds are shown as dashed lines.

The two structure types are very closely related to each other and are modifications of a basic tetra­hedral–octa­hedral framework structure (Figs. 2 ▸–4 ▸ ▸) containing inter­penetrating channels, which host the *M* ^+^ cations. The general building unit in these structure types contains *M* ^3+^O_6_ octa­hedra, which are connected *via* their six corners to six protonated AsO_4_ tetra­hedra (*M* ^3+^As_6_O_24_ group). These are in turn connected *via* three corners to other *M* ^3+^O_6_ octa­hedra, the free, protonated corner of each AsO_4_ tetra­hedron forming a hydrogen bond to the neighbouring *M* ^3+^As_6_O_24_ group. In both types, the *M* ^3+^As_6_O_24_ groups are arranged in layers perpendicular to the *c* axis (Fig. 2 ▸ *a*) and parallel to the *ab* plane (Fig. 3*a*). The groups within these layers are held together by medium-strong hydrogen bonds (Tables 2 ▸ and 3 ▸). The different modifications are caused by strong distortion of the whole structure (see detailed comparison in Lesage *et al.*, 2007 ▸).

**Figure 4:**
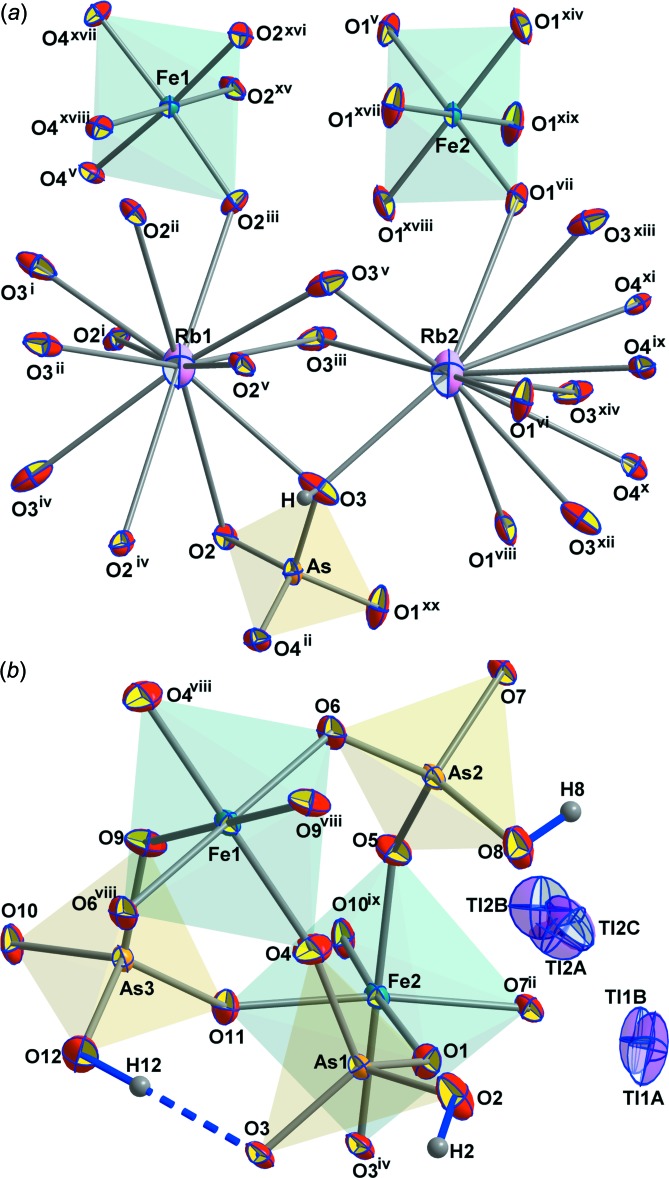
The principal building units of (*a*) RbFe(HAsO_4_)_2_ and (*b*) TlFe(HAsO_4_)_2_ shown as displacement ellipsoids at the 70% probability level. Symmetry codes: RbFe(HAsO_4_)_2_: (i) *x* − *y*, −*y*, −*z* + 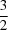; (ii) −*x*, −*x* + *y*, −*z* + 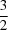; (iii) −*x* + *y*, −*x*, *z*; (iv) *y*, *x*, −*z* + 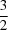; (v) −*y*, *x* − *y*, *z*; (vi) −*x* + 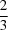, −*y* − 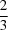, −*z* + 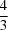; (vii) *y* + 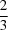, −*x* + *y*+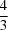, −*z* + 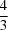; (viii) *x* − *y* − 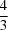, *x* − 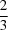, −*z* + 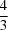; (ix) *x* − 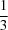, *x* − *y* − 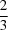, *z* − 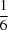; (*x*) −*y* − 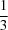, −*x* + 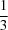, *z* − 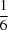; (xi) −*x* + *y* + 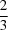, *y* + 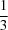, *z* − 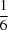; (xii) −*x* − 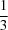, −*y* − 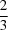, −*z* + 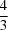; (xiii) *y* + 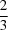, −*x* + *y* + 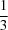, −*z* + 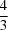; (xiv) *x* − *y* − 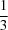, *x* + 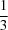, −*z* + 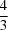; (xv) −*y*, *x* − *y* + 1, *z*; (xvi) *x* + 1, *y* + 1, *z*; (xvii) *x*, *y* + 1, *z*; (xviii) −*x* + *y* + 1, −*x* + 1, *z*; (xix) −*x* + 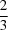, −*y* + 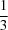, −*z* + 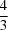; (xx) *x* − 1, *y*, *z;* TlFe(HAsO_4_)_2_: −*z*; (ii) −*x*, −*y* + 2, −*z*; (iv) −*x* + 1, −*y* + 1, −*z*; (viii) −*x*, −*y* + 1, −*z* + 1; (ix) −*x*, −*y* + 1, −*z*.

In both compounds the Tl/Rb atoms are 12-coordinated (Tables 4 ▸ and 5 ▸). The average Tl—O (3.279 and 3.312 Å) and Rb—O (3.257 and 3.390 Å) bond lengths are longer than the grand mean bond lengths in Tl/RbO_12_ polyhedra of 3.195 (Gagné & Hawthorne, 2018 ▸) and 3.228 Å (Gagné & Hawthorne, 2016 ▸), thus leading to rather low bond-valence sums (BVSs) (Gagné & Hawthorne, 2015 ▸) for the involved *M* ^+^ cations (0.76/0.88 and 0.82/0.85 valence units, v.u., for the RbFe and TlFe representative, respectively). The average Tl2—O bond length in TlFe(HAsO_4_)_2_ (3.312 Å) is the longest average bond length found so far for TlO_12_ polyhedra (max. Tl—O = 3.304 Å; Gagné & Hawthorne, 2018 ▸) and the corresponding average Rb2—O bond length in RbFe(HAsO_4_)_2_ is also close to the longest observed such bond lengths in RbO_12_ polyhedra of 3.410 Å (Gagné & Hawthorne, 2016 ▸). These loose bonds reflect the observation that the alkali cations ‘rattle’ somewhat in their hosting voids, with considerable positional disorder of the Tl atoms in these voids (Fig. 4 ▸ *b*). The Tl atoms were therefore modelled with two Tl1 positions (Tl1*A*, Tl1*B*) and three Tl2 positions (Tl2*A*, Tl2*B*, Tl2*C*), between 0.28 (2) and 0.48 (2) Å apart. The refined occupancies of the dominant positions (Tl1*A* and Tl2*A*) are 63 and 45%, respectively. The influence of a stereochemically active lone pair of electrons on the Tl^+^ cations may also play a role in the positional disorder.

The average Fe—O bond lengths, which show a fairly narrow range between 1.998 and 2.006 Å for the four FeO_6_ octa­hedra in the two title compounds, are slightly lower than the corresponding grand mean average of 2.011 Å reported by Baur (1981 ▸), thus leading to slightly higher BVSs of between 3.11 and 3.15 v.u. (Gagné & Hawthorne, 2015 ▸).

The AsO_4_ tetra­hedra are distorted with three short bond lengths of those bonds connecting to neighbouring FeO_6_ octa­hedra and one considerably elongated bond length to the protonated corner. The average As—O bond lengths are close to the calculated average of 1.686 (10) Å (calculated on 704 AsO_4_ polyhedra; Schwendtner, 2008 ▸), and the two As—OH bond lengths (Tables 3 ▸ and 4 ▸) are also close to the average of such lengths in HAsO_4_ polyhedra of 1.72 (3) Å (Schwendtner, 2008 ▸), but the two bond lengths to O atoms with rather strong hydrogen bonds [*D*⋯*A* = 2.569 (3) and 2.615 (3) Å] are considerably elongated to 1.738 (2) and 1.742 (2) Å, respectively (Tables 2 ▸ and 3 ▸).

## Synthesis and crystallization   

The compounds were grown by hydro­thermal synthesis at 493 K (7 d, autogeneous pressure, slow furnace cooling) using Teflon-lined stainless steel autoclaves with an approximate filling volume of 2 cm^3^. Reagent-grade Rb_2_CO_3_/Tl_2_CO_3_, Fe_2_O_3_ and H_3_AsO_4_·0.5H_2_O were used as starting reagents in approximate volume ratios of *M* ^+^:*M* ^3+^:As of 1:1:2. The vessels were filled with distilled water to about 70% of their inner volumes which led to initial and final pH values of 1.5 and 1, respectively, for both synthesis batches. The reaction products were washed thoroughly with distilled water, filtered and dried at room temperature. They are stable in air.

RbFe(HAsO_4_)_2_ formed colorless pseudohexa­gonal platelets (Fig. 1 ▸ *a*). TlFe(HAsO_4_)_2_ formed pseudo-‘disphenoidic-monoclinic’, short prismatic, colourless glassy crystals (Fig. 1 ▸ *b*), some of which showed fine-grained red inclusions, probably either unreacted Fe_2_O_3_ or some Fe–O–(OH) compound, mainly in the core of the crystals.

Measured X-ray powder diffraction diagrams of RbFe(HAsO_4_)_2_ and TlFe(HAsO_4_)_2_ were deposited at the Inter­national Centre for Diffraction Data under PDF numbers 00-057-0160 (Prem *et al.*, 2005*a* ▸) and 00-057-0159 (Prem *et al.*, 2005*b* ▸), respectively.

The chemical compositions of the title compounds were checked by standard SEM–EDS analysis of several carbon-coated crystals of each compound; no impurities could be detected.

## Refinement   

Crystal data, data collection and structure refinement details are summarized in Table 6 ▸.

For the final refinement the atomic positions of RbFe(HPO_4_)_2_ (Lii & Wu, 1994 ▸) and CsSc(HAsO_4_)_2_ (Schwendtner & Kolitsch, 2004 ▸) were used for RbFe(HAsO_4_)_2_ and TlFe(HAsO_4_)_2_, respectively. The H atoms were then located from the difference-Fourier map and O—H distances were restrained to 0.90 (4) Å. The position of H8 was fixed to the coordinates where it was located in the difference-Fourier map, since a refinement of the position led to an unreasonably close distance to the neighbouring As atom. At this point, electron densities of up to 2.79 and 4.71 e Å^−3^, respectively, were found close to the Tl1 and Tl2 atoms, along with anomalous displacement ellipsoids of these atoms. This suggested the presence of positional disorder (and, possibly, some mobility) of the Tl atoms in the cavities. The disorder was then modeled by additional, partially occupied Tl positions. The bulk occupancy for each of the two disordered Tl positions (Tl1*A* and Tl1*B* for Tl1 and Tl2*A*, Tl2*B* and Tl2*C* for Tl2) was constrained to 1.00. As a result, the *R* value dropped from 0.0335 to 0.0224, and the weight parameters also improved. Final equivalent isotropic displacement parameters of all the partially occupied Tl sites are reasonable, with values between *ca* 0.03 and 0.04 Å^2^, very similar to those in the Rb compound. The final residual electron densities are < 1 e Å^−3^ for both compounds.

## Supplementary Material

Crystal structure: contains datablock(s) RbFeHAsO42, TlFeHAsO42. DOI: 10.1107/S2056989018006473/pj2051sup1.cif

Structure factors: contains datablock(s) RbFeHAsO42. DOI: 10.1107/S2056989018006473/pj2051RbFeHAsO42sup3.hkl

Structure factors: contains datablock(s) TlFeHAsO42. DOI: 10.1107/S2056989018006473/pj2051TlFeHAsO42sup2.hkl

CCDC references: 1839860, 1839859

Additional supporting information:  crystallographic information; 3D view; checkCIF report

## Figures and Tables

**Table 1:** Compilation of all published compounds adopting the (NH_4_)Fe(HPO_4_)_2_ structure type (Yakubovich, 1993 ▸) and the RbFe(HPO_4_)_2_ structure type (Lii & Wu, 1994 ▸)

(NH_4_)Fe(HPO_4_)_2_ type (*P* 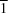 , *Z* = 3)							
	*a* (Å)	*b* (Å)	*c* (Å)	*α* (°)	*β* (°)	*γ* (°)	*V* (Å^3^)
CsSc(HAsO_4_)_2_^*a*^	7.520 (2)	9.390 (2)	10.050 (2)	65.48 (3)	70.66 (3)	70.10 (3)	592.0 (2)
TlFe(HAsO_4_)_2_	7.346 (2)	9.148 (2)	9.662 (2)	64.89 (3)	70.51 (3)	69.94 (3)	538.6 (2)
(NH_4_)Fe(HAsO_4_)_2_^*b*^	7.3473 (7)	9.1917 (8)	9.7504 (9)	64.545 (5)	70.710 (7)	69.638 (6)	544.54 (2)
(NH_4_)Fe(HPO_4_)_2_^*c*^	7.185 (3)	8.857 (3)	9.478 (3)	64.79 (3)	70.20 (3)	69.38 (3)	498.0 (3)
(NH_4_)Fe(HPO_4_)_2_^*d*^	7.121	8.839	9.465	64.598	70.321	69.574	491.88
(NH_4_)V(HPO_4_)_2_^*e*^	7.173 (2)	8.841 (2)	9.458 (2)	65.08 (2)	70.68 (2)	69.59 (2)	497.59 (2)
(NH_4_)(Al_0.64_Ga_0.36_)^*f*^(HPO_4_)_2_	7.109 (4)	8.695 (4)	9.252 (6)	65.01 (4)	70.25 (5)	69.01 (4)	472.1 (4)
(ND_4_)Fe(DPO_4_)_2_^*d*^,^*g*^	7.11830 (3)	8.83828 (4)	9.46407 (4)	64.5802 (4)	70.3127 (4)	69.5733 (5)	491.495 (4)
KFe(HPO_4_)_2_^*h*^	7.20	8.76	9.49	64.58	69.82	70.13	
(H_3_O)Al(HPO_4_)_2_^*i*^	7.1177 (2)	8.6729 (2)	9.2200 (3)	65.108 (2)	70.521 (1)	68.504 (2)	469.4 (2)
CsIn(HPO_4_)_2_^*j*^	7.4146 (3)	9.0915 (3)	9.7849 (3)	65.525 (3)	70.201 (3)	69.556 (3)	547.77 (4)
RbFe(HPO_4_)_2_^*j*^	7.2025 (4)	8.8329 (8)	9.4540 (8)	65.149 (8)	70.045 (6)	69.591 (6)	497.44 (8)
RbV(HPO_4_)_2_^*k*^	7.188 (2)	8.831 (1)	9.450(2	65.34	70.449	69.739	498.5 (2)
RbFe(HPO_4_)_2_ type (*R* 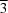 *c*, *Z* = 18)							
RbIn(HAsO_4_)_2_^*l*^	8.512 (1)	8.512 (1)	56.43 (1)	90	90	120	3541.1 (9)
CsIn(HAsO_4_)_2_^*l*^	8.629 (1)	8.629 (1)	56.99 (1)	90	90	120	3674.7 (9)
RbAl(HAsO_4_)_2_^*m*^	8.318 (1)	8.318 (1)	52.87 (1)	90	90	120	3167.9 (9)
RbFe(HAsO_4_)_2_	8.425 (1)	8.425 (1)	54.75 (1)	90	90	120	3365.5 (9)
CsFe(HAsO_4_)_2_^*m*^	8.525 (1)	8.525 (1)	55.00 (1)	90	90	120	3461.5 (9)
RbFe(HPO_4_)_2_^*n*^	8.160 (1)	8.160 (1)	52.75 (1)	90	90	120	3041.82
RbAl(HPO_4_)_2_^*j*^	8.0581 (18)	8.0581 (18)	51.081 (12)	90	90	120	2872 (11)
RbGa(HPO_4_)_2_^*j*^	8.1188 (15)	8.1188 (15)	51.943 (4)	90	90	120	2965.1 (8)

Notes: (*a*) Schwendtner & Kolitsch (2004 ▸); (*b*) Ouerfelli *et al.* (2014 ▸); (*c*) Yakubovich (1993 ▸), transformed from *I* 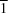; (*d*) Alfonso *et al.* (2011 ▸), converted to reduced cell; (*e*) Bircsak & Harrison (1998 ▸); (*f*) Stalder & Wilkinson (1998 ▸); (*g*) Alfonso *et al.* (2010 ▸); (*h*) Smith & Brown (1959 ▸); (*i*) Yan *et al.* (2000 ▸); (*j*) Lesage *et al.* (2007 ▸); (*k*) Haushalter *et al.* (1995 ▸), converted to reduced cell; (*l*) Schwendtner & Kolitsch (2017 ▸); (*m*) Schwendtner & Kolitsch (2018 ▸); (*n*) Lii & Wu (1994 ▸).

**Table 2:** Hydrogen-bond geometry (Å, °) for RbFe(HAsO_4_)_2_

*D*—H⋯*A*	*D*—H	H⋯*A*	*D*⋯*A*	*D*—H⋯*A*
O3—H⋯O4^xxi^	0.81 (3)	1.82 (3)	2.615 (3)	166 (4)

Symmetry code: (xxi) 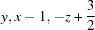.

**Table 3:** Hydrogen-bond geometry (Å, °) for TlFe(HAsO_4_)_2_

*D*—H⋯*A*	*D*—H	H⋯*A*	*D*⋯*A*	*D*—H⋯*A*
O2—H2⋯O9^iii^	0.85 (3)	1.86 (3)	2.707 (3)	176 (5)
O8—H8⋯O10^v^	0.982 (2)	1.598 (2)	2.569 (3)	169.44 (15)
O12—H12⋯O3	0.88 (3)	1.86 (3)	2.729 (3)	172 (5)

Symmetry codes: (iii) 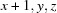; (v) 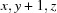.

**Table 4:** Selected bond lengths (Å) for RbFe(HAsO_4_)_2_

Rb1—O3	3.146 (2)	Rb2—O4^xi^	3.562 (2)
Rb1—O3^i^	3.147 (2)	Rb2—O3^xii^	3.640 (2)
Rb1—O3^ii^	3.147 (2)	Rb2—O3^xiii^	3.640 (2)
Rb1—O3^iii^	3.147 (2)	Rb2—O3^xiv^	3.640 (2)
Rb1—O3^iv^	3.147 (2)	Fe1—O2^xv^	1.9957 (18)
Rb1—O3^v^	3.147 (2)	Fe1—O2^iii^	1.9957 (18)
Rb1—O2^ii^	3.3671 (19)	Fe1—O2^xvi^	1.9957 (18)
Rb1—O2^iv^	3.3671 (19)	Fe1—O4^xvii^	2.0055 (19)
Rb1—O2^iii^	3.3671 (19)	Fe1—O4^v^	2.0055 (18)
Rb1—O2^i^	3.3671 (19)	Fe1—O4^xviii^	2.0055 (18)
Rb1—O2^v^	3.3671 (19)	Fe2—O1^vii^	1.998 (2)
Rb1—O2	3.3671 (19)	Fe2—O1^xiv^	1.998 (2)
Rb2—O3^v^	2.965 (2)	Fe2—O1^xix^	1.998 (2)
Rb2—O3^iii^	2.965 (2)	Fe2—O1^v^	1.998 (2)
Rb2—O3	2.965 (2)	Fe2—O1^xviii^	1.998 (2)
Rb2—O1^vi^	3.394 (2)	Fe2—O1^xvii^	1.998 (2)
Rb2—O1^vii^	3.394 (2)	As—O1^xx^	1.6555 (19)
Rb2—O1^viii^	3.394 (2)	As—O2	1.6720 (18)
Rb2—O4^ix^	3.562 (2)	As—O4^ii^	1.6801 (18)
Rb2—O4^x^	3.562 (2)	As—O3	1.742 (2)

Symmetry codes: (i) 
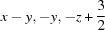
; (ii) 
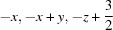
; (iii) 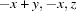; (iv) 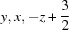; (v) 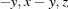; (vi) 
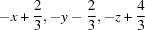
; (vii) 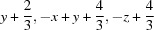; (viii) 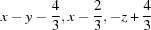; (ix) 
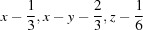
; (x) 
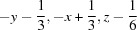
; (xi) 
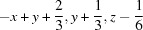
; (xii) 
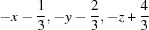
; (xiii) 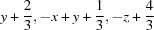; (xiv) 
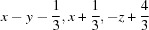
; (xv) 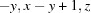; (xvi) 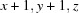; (xvii) 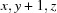; (xviii) 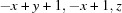; (xix) 
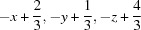
; (xx) 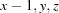.

**Table 5:** Selected bond lengths (Å) for TlFe(HAsO_4_)_2_

Tl1*A*—O1	2.853 (2)	Fe1—O4^viii^	1.942 (2)
Tl1*A*—O1^i^	2.853 (2)	Fe1—O4	1.942 (2)
Tl1*A*—O8^i^	3.094 (3)	Fe1—O6^viii^	2.015 (2)
Tl1*A*—O8	3.094 (3)	Fe1—O6	2.015 (2)
Tl1*A*—O2	3.227 (3)	Fe1—O9	2.060 (2)
Tl1*A*—O2^i^	3.227 (3)	Fe1—O9^viii^	2.060 (2)
Tl1*A*—O7^ii^	3.344 (2)	Fe2—O5	1.946 (2)
Tl1*A*—O7^iii^	3.344 (2)	Fe2—O11	1.970 (2)
Tl1*A*—O5^ii^	3.543 (2)	Fe2—O1	1.978 (2)
Tl1*A*—O5^iii^	3.543 (2)	Fe2—O10^ix^	2.014 (2)
Tl1*A*—O12^iv^	3.615 (3)	Fe2—O7^ii^	2.044 (2)
Tl1*A*—O12^v^	3.615 (3)	Fe2—O3^iv^	2.065 (2)
Tl2*A*—O3^vi^	2.804 (4)	As1—O4	1.652 (2)
Tl2*A*—O2	2.852 (4)	As1—O1	1.668 (2)
Tl2*A*—O6^iii^	2.936 (5)	As1—O3	1.683 (2)
Tl2*A*—O12^v^	3.020 (4)	As1—O2	1.720 (2)
Tl2*A*—O8	3.091 (5)	As2—O6	1.670 (2)
Tl2*A*—O7^iii^	3.362 (5)	As2—O5	1.671 (2)
Tl2*A*—O7^vii^	3.450 (4)	As2—O7	1.684 (2)
Tl2*A*—O9^viii^	3.523 (5)	As2—O8	1.738 (2)
Tl2*A*—O10^viii^	3.572 (5)	As3—O11	1.655 (2)
Tl2*A*—O12^vi^	3.638 (5)	As3—O10	1.6730 (19)
Tl2*A*—O4^vi^	3.691 (4)	As3—O9	1.679 (2)
Tl2*A*—O4	3.811 (5)	As3—O12	1.721 (2)

Symmetry codes: (i) 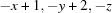; (ii) 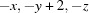; (iii) 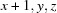; (iv) 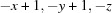; (v) 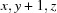; (vi) 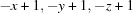; (vii) 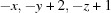; (viii) 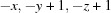; (ix) 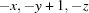.

**Table 6:** Experimental details

	RbFe(HAsO_4_)_2_	TlFe(HAsO_4_)_2_
Crystal data		
*M*_r_	421.18	540.08
Crystal system, space group	Trigonal, *R* 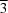 *c*:*H*	Triclinic, *P* 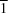
Temperature (K)	293	293
*a*, *b*, *c* (Å)	8.425 (1), 8.425 (1), 54.749 (11)	7.346 (2), 9.148 (2), 9.662 (2)
α, β, γ (°)	90, 90, 120	64.89 (3), 70.51 (3), 69.94 (3)
*V* (Å^3^)	3365.5 (10)	538.6 (3)
*Z*	18	3
Radiation type	Mo *K*α	Mo *K*α
μ (mm^−1^)	17.27	33.58
Crystal size (mm)	0.09 × 0.08 × 0.03	0.10 × 0.05 × 0.04

Data collection		
Diffractometer	Nonius KappaCCD single-crystal four-circle	Nonius KappaCCD single-crystal four-circle
Absorption correction	Multi-scan (*HKL* *SCALEPACK*; Otwinowski *et al.*, 2003 ▸)	Multi-scan (*HKL* *SCALEPACK*; Otwinowski *et al.*, 2003 ▸)
*T*_min_, *T*_max_	0.306, 0.625	0.134, 0.347
No. of measured, independent and observed [*I* > 2σ(*I*)] reflections	3994, 1105, 1014	7723, 3906, 3391
*R*_int_	0.023	0.021
(sin θ/λ)_max_ (Å^−1^)	0.704	0.758

Refinement		
*R*[*F* ^2^ > 2σ(*F* ^2^)], *wR*(*F* ^2^), *S*	0.021, 0.052, 1.12	0.022, 0.051, 1.06
No. of reflections	1105	3906
No. of parameters	62	208
No. of restraints	1	4
H-atom treatment	All H-atom parameters refined	Only H-atom displacement parameters refined
Δρ_max_, Δρ_min_ (e Å^−3^)	0.87, −0.72	0.96, −1.13

Computer programs: *COLLECT* (Nonius, 2003 ▸), *HKL* *DENZO* and *SCALEPACK* (Otwinowski *et al.*, 2003 ▸), *SHELXS97* (Sheldrick, 2008 ▸), *SHELXL2016* (Sheldrick, 2015 ▸), *DIAMOND* (Brandenburg, 2005 ▸), *publCIF* (Westrip, 2010 ▸) and *WinGX* (Farrugia, 2012 ▸).

## supplementary crystallographic information

### Rubidium iron bis[hydrogen arsenate(V)] (RbFeHAsO42) Crystal data

**Table:**

RbFe(HAsO_4_)_2_	*D*_x_ = 3.741 Mg m^−^^3^
*M**_r_* = 421.18	Mo *K*α radiation, λ = 0.71073 Å
Trigonal, *R*3*c*:*H*	Cell parameters from 2.794 reflections
*a* = 8.425 (1) Å	θ = 2.9–30.0°
*c* = 54.749 (11) Å	µ = 17.27 mm^−^^1^
*V* = 3365.5 (10) Å^3^	*T* = 293 K
*Z* = 18	Hexagonal platelet, colourless
*F*(000) = 3510	0.09 × 0.08 × 0.03 mm

### Rubidium iron bis[hydrogen arsenate(V)] (RbFeHAsO42) Data collection

**Table:**

Nonius KappaCCD single-crystal four-circle diffractometer	1014 reflections with *I* > 2σ(*I*)
Radiation source: fine-focus sealed tube	*R*_int_ = 0.023
φ and ω scans	θ_max_ = 30.0°, θ_min_ = 2.9°
Absorption correction: multi-scan (HKL SCALEPACK; Otwinowski *et al.*, 2003)	*h* = −11→11
*T*_min_ = 0.306, *T*_max_ = 0.625	*k* = −9→9
3994 measured reflections	*l* = −76→76
1105 independent reflections	

### Rubidium iron bis[hydrogen arsenate(V)] (RbFeHAsO42) Refinement

**Table:**

Refinement on *F*^2^	Secondary atom site location: difference Fourier map
Least-squares matrix: full	Hydrogen site location: difference Fourier map
*R*[*F*^2^ > 2σ(*F*^2^)] = 0.021	All H-atom parameters refined
*wR*(*F*^2^) = 0.052	*w* = 1/[σ^2^(*F*_o_^2^) + (0.0241*P*)^2^ + 19.8694*P*] where *P* = (*F*_o_^2^ + 2*F*_c_^2^)/3
*S* = 1.12	(Δ/σ)_max_ = 0.001
1105 reflections	Δρ_max_ = 0.87 e Å^−^^3^
62 parameters	Δρ_min_ = −0.72 e Å^−^^3^
1 restraint	Extinction correction: SHELXL2016 (Sheldrick, 2015), Fc^*^=kFc[1+0.001xFc^2^λ^3^/sin(2θ)]^-1/4^
Primary atom site location: structure-invariant direct methods	Extinction coefficient: 0.000112 (19)

### Rubidium iron bis[hydrogen arsenate(V)] (RbFeHAsO42) Special details

**Table:**

Geometry. All esds (except the esd in the dihedral angle between two l.s. planes) are estimated using the full covariance matrix. The cell esds are taken into account individually in the estimation of esds in distances, angles and torsion angles; correlations between esds in cell parameters are only used when they are defined by crystal symmetry. An approximate (isotropic) treatment of cell esds is used for estimating esds involving l.s. planes.

### Rubidium iron bis[hydrogen arsenate(V)] (RbFeHAsO42) Fractional atomic coordinates and isotropic or equivalent isotropic displacement parameters (Å^2^)

**Table:**

	*x*	*y*	*z*	*U*_iso_*/*U*_eq_	
Rb1	0.000000	0.000000	0.750000	0.03271 (19)	
Rb2	0.000000	0.000000	0.66752 (2)	0.03704 (16)	
Fe1	0.333333	0.666667	0.75352 (2)	0.00828 (13)	
Fe2	0.333333	0.666667	0.666667	0.00999 (17)	
As	−0.42107 (3)	−0.38770 (3)	0.71298 (2)	0.00949 (9)	
O1	0.4739 (3)	−0.4215 (3)	0.68632 (3)	0.0215 (4)	
O2	−0.4425 (2)	−0.2504 (2)	0.73312 (3)	0.0123 (3)	
O3	−0.1873 (3)	−0.2762 (3)	0.70652 (4)	0.0200 (4)	
O4	0.4749 (2)	−0.1197 (2)	0.77593 (3)	0.0117 (3)	
H	−0.149 (5)	−0.342 (4)	0.7113 (6)	0.022 (10)*	

### Rubidium iron bis[hydrogen arsenate(V)] (RbFeHAsO42) Atomic displacement parameters (Å^2^)

**Table:**

	*U*^11^	*U*^22^	*U*^33^	*U*^12^	*U*^13^	*U*^23^
Rb1	0.0391 (3)	0.0391 (3)	0.0199 (4)	0.01955 (14)	0.000	0.000
Rb2	0.0453 (2)	0.0453 (2)	0.0205 (3)	0.02266 (12)	0.000	0.000
Fe1	0.00900 (18)	0.00900 (18)	0.0068 (3)	0.00450 (9)	0.000	0.000
Fe2	0.0122 (3)	0.0122 (3)	0.0055 (4)	0.00610 (13)	0.000	0.000
As	0.01225 (14)	0.01058 (13)	0.00780 (13)	0.00733 (10)	0.00099 (9)	0.00115 (9)
O1	0.0324 (12)	0.0306 (11)	0.0101 (8)	0.0222 (10)	−0.0067 (8)	−0.0012 (8)
O2	0.0127 (8)	0.0115 (8)	0.0126 (8)	0.0060 (7)	0.0039 (7)	−0.0015 (7)
O3	0.0153 (9)	0.0184 (10)	0.0295 (11)	0.0110 (8)	0.0104 (8)	0.0129 (8)
O4	0.0128 (8)	0.0108 (8)	0.0138 (8)	0.0076 (7)	−0.0019 (7)	−0.0048 (7)

### Rubidium iron bis[hydrogen arsenate(V)] (RbFeHAsO42) Geometric parameters (Å, º)

**Table:**

Rb1—O3	3.146 (2)	Rb2—O3^xii^	3.640 (2)
Rb1—O3^i^	3.147 (2)	Rb2—O3^xiii^	3.640 (2)
Rb1—O3^ii^	3.147 (2)	Rb2—O3^xiv^	3.640 (2)
Rb1—O3^iii^	3.147 (2)	Rb2—As^xii^	3.8044 (6)
Rb1—O3^iv^	3.147 (2)	Rb2—As^xiii^	3.8044 (6)
Rb1—O3^v^	3.147 (2)	Rb2—As^xiv^	3.8044 (6)
Rb1—O2^ii^	3.3671 (19)	Fe1—O2^xv^	1.9957 (18)
Rb1—O2^iv^	3.3671 (19)	Fe1—O2^iii^	1.9957 (18)
Rb1—O2^iii^	3.3671 (19)	Fe1—O2^xvi^	1.9957 (18)
Rb1—O2^i^	3.3671 (19)	Fe1—O4^xvii^	2.0055 (19)
Rb1—O2^v^	3.3671 (19)	Fe1—O4^v^	2.0055 (18)
Rb1—O2	3.3671 (19)	Fe1—O4^xviii^	2.0055 (18)
Rb1—H	3.28 (3)	Fe2—O1^vii^	1.998 (2)
Rb1—H^v^	3.28 (4)	Fe2—O1^xiv^	1.998 (2)
Rb1—H^iii^	3.28 (3)	Fe2—O1^xix^	1.998 (2)
Rb2—O3^v^	2.965 (2)	Fe2—O1^v^	1.998 (2)
Rb2—O3^iii^	2.965 (2)	Fe2—O1^xviii^	1.998 (2)
Rb2—O3	2.965 (2)	Fe2—O1^xvii^	1.998 (2)
Rb2—O1^vi^	3.394 (2)	As—O1^xx^	1.6555 (19)
Rb2—O1^vii^	3.394 (2)	As—O2	1.6720 (18)
Rb2—O1^viii^	3.394 (2)	As—O4^ii^	1.6801 (18)
Rb2—O4^ix^	3.562 (2)	As—O3	1.742 (2)
Rb2—O4^x^	3.562 (2)	O3—H	0.81 (3)
Rb2—O4^xi^	3.562 (2)		
			
O3—Rb1—O3^i^	164.86 (8)	O4^xi^—Rb2—As^xii^	70.60 (3)
O3—Rb1—O3^ii^	121.47 (8)	O3^xii^—Rb2—As^xii^	26.95 (3)
O3^i^—Rb1—O3^ii^	68.99 (6)	O3^xiii^—Rb2—As^xii^	62.25 (4)
O3—Rb1—O3^iii^	68.99 (6)	O3^xiv^—Rb2—As^xii^	96.36 (4)
O3^i^—Rb1—O3^iii^	103.28 (7)	O3^v^—Rb2—As^xiii^	101.27 (5)
O3^ii^—Rb1—O3^iii^	164.86 (8)	O3^iii^—Rb2—As^xiii^	105.92 (5)
O3—Rb1—O3^iv^	103.28 (8)	O3—Rb2—As^xiii^	175.05 (4)
O3^i^—Rb1—O3^iv^	68.99 (6)	O1^vi^—Rb2—As^xiii^	88.88 (4)
O3^ii^—Rb1—O3^iv^	68.99 (6)	O1^vii^—Rb2—As^xiii^	25.79 (3)
O3^iii^—Rb1—O3^iv^	121.47 (8)	O1^viii^—Rb2—As^xiii^	104.27 (4)
O3—Rb1—O3^v^	68.99 (6)	O4^ix^—Rb2—As^xiii^	53.64 (3)
O3^i^—Rb1—O3^v^	121.47 (8)	O4^x^—Rb2—As^xiii^	70.60 (3)
O3^ii^—Rb1—O3^v^	103.28 (7)	O4^xi^—Rb2—As^xiii^	26.10 (3)
O3^iii^—Rb1—O3^v^	68.99 (6)	O3^xii^—Rb2—As^xiii^	96.36 (4)
O3^iv^—Rb1—O3^v^	164.86 (8)	O3^xiii^—Rb2—As^xiii^	26.95 (3)
O3—Rb1—O2^ii^	126.04 (5)	O3^xiv^—Rb2—As^xiii^	62.24 (4)
O3^i^—Rb1—O2^ii^	68.90 (5)	As^xii^—Rb2—As^xiii^	78.998 (15)
O3^ii^—Rb1—O2^ii^	48.72 (5)	O3^v^—Rb2—As^xiv^	175.05 (4)
O3^iii^—Rb1—O2^ii^	116.78 (5)	O3^iii^—Rb2—As^xiv^	101.27 (5)
O3^iv^—Rb1—O2^ii^	113.39 (5)	O3—Rb2—As^xiv^	105.92 (5)
O3^v^—Rb1—O2^ii^	65.41 (5)	O1^vi^—Rb2—As^xiv^	104.27 (4)
O3—Rb1—O2^iv^	65.40 (5)	O1^vii^—Rb2—As^xiv^	88.88 (4)
O3^i^—Rb1—O2^iv^	113.39 (5)	O1^viii^—Rb2—As^xiv^	25.79 (3)
O3^ii^—Rb1—O2^iv^	68.90 (5)	O4^ix^—Rb2—As^xiv^	70.60 (3)
O3^iii^—Rb1—O2^iv^	126.04 (5)	O4^x^—Rb2—As^xiv^	26.10 (3)
O3^iv^—Rb1—O2^iv^	48.72 (5)	O4^xi^—Rb2—As^xiv^	53.64 (3)
O3^v^—Rb1—O2^iv^	116.78 (5)	O3^xii^—Rb2—As^xiv^	62.24 (4)
O2^ii^—Rb1—O2^iv^	112.77 (3)	O3^xiii^—Rb2—As^xiv^	96.36 (4)
O3—Rb1—O2^iii^	113.39 (5)	O3^xiv^—Rb2—As^xiv^	26.95 (3)
O3^i^—Rb1—O2^iii^	65.41 (5)	As^xii^—Rb2—As^xiv^	78.998 (15)
O3^ii^—Rb1—O2^iii^	116.78 (5)	As^xiii^—Rb2—As^xiv^	78.997 (15)
O3^iii^—Rb1—O2^iii^	48.72 (5)	O2^xv^—Fe1—O2^iii^	91.74 (8)
O3^iv^—Rb1—O2^iii^	126.04 (5)	O2^xv^—Fe1—O2^xvi^	91.74 (8)
O3^v^—Rb1—O2^iii^	68.90 (5)	O2^iii^—Fe1—O2^xvi^	91.74 (8)
O2^ii^—Rb1—O2^iii^	74.90 (6)	O2^xv^—Fe1—O4^xvii^	92.04 (8)
O2^iv^—Rb1—O2^iii^	171.63 (6)	O2^iii^—Fe1—O4^xvii^	175.92 (8)
O3—Rb1—O2^i^	116.78 (5)	O2^xvi^—Fe1—O4^xvii^	89.66 (7)
O3^i^—Rb1—O2^i^	48.72 (5)	O2^xv^—Fe1—O4^v^	89.66 (7)
O3^ii^—Rb1—O2^i^	113.39 (5)	O2^iii^—Fe1—O4^v^	92.04 (8)
O3^iii^—Rb1—O2^i^	65.41 (5)	O2^xvi^—Fe1—O4^v^	175.92 (8)
O3^iv^—Rb1—O2^i^	68.90 (5)	O4^xvii^—Fe1—O4^v^	86.47 (8)
O3^v^—Rb1—O2^i^	126.04 (5)	O2^xv^—Fe1—O4^xviii^	175.92 (8)
O2^ii^—Rb1—O2^i^	112.77 (3)	O2^iii^—Fe1—O4^xviii^	89.66 (7)
O2^iv^—Rb1—O2^i^	112.77 (3)	O2^xvi^—Fe1—O4^xviii^	92.04 (7)
O2^iii^—Rb1—O2^i^	59.80 (6)	O4^xvii^—Fe1—O4^xviii^	86.47 (8)
O3—Rb1—O2^v^	68.90 (5)	O4^v^—Fe1—O4^xviii^	86.47 (8)
O3^i^—Rb1—O2^v^	126.04 (5)	O2^xv^—Fe1—Rb2^xxi^	124.02 (5)
O3^ii^—Rb1—O2^v^	65.41 (5)	O2^iii^—Fe1—Rb2^xxi^	124.02 (6)
O3^iii^—Rb1—O2^v^	113.39 (5)	O2^xvi^—Fe1—Rb2^xxi^	124.02 (5)
O3^iv^—Rb1—O2^v^	116.78 (5)	O4^xvii^—Fe1—Rb2^xxi^	52.27 (6)
O3^v^—Rb1—O2^v^	48.72 (5)	O4^v^—Fe1—Rb2^xxi^	52.27 (5)
O2^ii^—Rb1—O2^v^	59.80 (6)	O4^xviii^—Fe1—Rb2^xxi^	52.27 (5)
O2^iv^—Rb1—O2^v^	74.90 (6)	O1^vii^—Fe2—O1^xiv^	93.72 (8)
O2^iii^—Rb1—O2^v^	112.77 (3)	O1^vii^—Fe2—O1^xix^	93.72 (8)
O2^i^—Rb1—O2^v^	171.63 (7)	O1^xiv^—Fe2—O1^xix^	93.72 (8)
O3—Rb1—O2	48.71 (5)	O1^vii^—Fe2—O1^v^	180.0
O3^i^—Rb1—O2	116.78 (5)	O1^xiv^—Fe2—O1^v^	86.29 (8)
O3^ii^—Rb1—O2	126.04 (5)	O1^xix^—Fe2—O1^v^	86.29 (8)
O3^iii^—Rb1—O2	68.90 (5)	O1^vii^—Fe2—O1^xviii^	86.29 (8)
O3^iv^—Rb1—O2	65.41 (5)	O1^xiv^—Fe2—O1^xviii^	180.0
O3^v^—Rb1—O2	113.39 (5)	O1^xix^—Fe2—O1^xviii^	86.29 (8)
O2^ii^—Rb1—O2	171.63 (6)	O1^v^—Fe2—O1^xviii^	93.71 (8)
O2^iv^—Rb1—O2	59.80 (6)	O1^vii^—Fe2—O1^xvii^	86.29 (8)
O2^iii^—Rb1—O2	112.77 (3)	O1^xiv^—Fe2—O1^xvii^	86.29 (8)
O2^i^—Rb1—O2	74.90 (6)	O1^xix^—Fe2—O1^xvii^	180.0
O2^v^—Rb1—O2	112.77 (3)	O1^v^—Fe2—O1^xvii^	93.71 (8)
O3—Rb1—H	14.3 (5)	O1^xviii^—Fe2—O1^xvii^	93.71 (8)
O3^i^—Rb1—H	169.7 (7)	O1^vii^—Fe2—Rb2^xix^	58.77 (7)
O3^ii^—Rb1—H	107.3 (5)	O1^xiv^—Fe2—Rb2^xix^	70.91 (7)
O3^iii^—Rb1—H	82.4 (5)	O1^xix^—Fe2—Rb2^xix^	146.11 (6)
O3^iv^—Rb1—H	100.7 (7)	O1^v^—Fe2—Rb2^xix^	121.23 (7)
O3^v^—Rb1—H	68.4 (6)	O1^xviii^—Fe2—Rb2^xix^	109.09 (7)
O2^ii^—Rb1—H	116.4 (6)	O1^xvii^—Fe2—Rb2^xix^	33.90 (6)
O2^iv^—Rb1—H	56.8 (6)	O1^vii^—Fe2—Rb2^xvii^	121.23 (7)
O2^iii^—Rb1—H	123.8 (6)	O1^xiv^—Fe2—Rb2^xvii^	109.09 (7)
O2^i^—Rb1—H	129.4 (6)	O1^xix^—Fe2—Rb2^xvii^	33.90 (6)
O2^v^—Rb1—H	57.1 (6)	O1^v^—Fe2—Rb2^xvii^	58.77 (7)
O2—Rb1—H	56.9 (6)	O1^xviii^—Fe2—Rb2^xvii^	70.91 (7)
O3—Rb1—H^v^	82.4 (5)	O1^xvii^—Fe2—Rb2^xvii^	146.10 (6)
O3^i^—Rb1—H^v^	107.3 (6)	Rb2^xix^—Fe2—Rb2^xvii^	180.0
O3^ii^—Rb1—H^v^	100.7 (6)	O1^vii^—Fe2—Rb2^xvi^	109.09 (7)
O3^iii^—Rb1—H^v^	68.4 (7)	O1^xiv^—Fe2—Rb2^xvi^	33.90 (6)
O3^iv^—Rb1—H^v^	169.7 (6)	O1^xix^—Fe2—Rb2^xvi^	121.23 (7)
O3^v^—Rb1—H^v^	14.3 (5)	O1^v^—Fe2—Rb2^xvi^	70.91 (7)
O2^ii^—Rb1—H^v^	56.8 (6)	O1^xviii^—Fe2—Rb2^xvi^	146.10 (6)
O2^iv^—Rb1—H^v^	129.4 (6)	O1^xvii^—Fe2—Rb2^xvi^	58.77 (7)
O2^iii^—Rb1—H^v^	57.1 (6)	Rb2^xix^—Fe2—Rb2^xvi^	60.0
O2^i^—Rb1—H^v^	116.4 (6)	Rb2^xvii^—Fe2—Rb2^xvi^	120.0
O2^v^—Rb1—H^v^	56.9 (6)	O1^vii^—Fe2—Rb2	33.90 (6)
O2—Rb1—H^v^	123.8 (6)	O1^xiv^—Fe2—Rb2	121.23 (7)
H—Rb1—H^v^	82.7 (9)	O1^xix^—Fe2—Rb2	109.09 (7)
O3—Rb1—H^iii^	68.5 (6)	O1^v^—Fe2—Rb2	146.10 (6)
O3^i^—Rb1—H^iii^	100.7 (6)	O1^xviii^—Fe2—Rb2	58.77 (7)
O3^ii^—Rb1—H^iii^	169.7 (6)	O1^xvii^—Fe2—Rb2	70.91 (7)
O3^iii^—Rb1—H^iii^	14.3 (5)	Rb2^xix^—Fe2—Rb2	60.0
O3^iv^—Rb1—H^iii^	107.3 (5)	Rb2^xvii^—Fe2—Rb2	120.0
O3^v^—Rb1—H^iii^	82.4 (5)	Rb2^xvi^—Fe2—Rb2	120.0
O2^ii^—Rb1—H^iii^	129.4 (6)	O1^vii^—Fe2—Rb2^xxii^	70.91 (7)
O2^iv^—Rb1—H^iii^	116.4 (7)	O1^xiv^—Fe2—Rb2^xxii^	146.11 (6)
O2^iii^—Rb1—H^iii^	56.9 (6)	O1^xix^—Fe2—Rb2^xxii^	58.77 (7)
O2^i^—Rb1—H^iii^	56.8 (6)	O1^v^—Fe2—Rb2^xxii^	109.09 (7)
O2^v^—Rb1—H^iii^	123.8 (6)	O1^xviii^—Fe2—Rb2^xxii^	33.90 (6)
O2—Rb1—H^iii^	57.1 (6)	O1^xvii^—Fe2—Rb2^xxii^	121.23 (7)
H—Rb1—H^iii^	82.7 (9)	Rb2^xix^—Fe2—Rb2^xxii^	120.0
H^v^—Rb1—H^iii^	82.7 (9)	Rb2^xvii^—Fe2—Rb2^xxii^	60.0
O3^v^—Rb2—O3^iii^	73.88 (7)	Rb2^xvi^—Fe2—Rb2^xxii^	180.0
O3^v^—Rb2—O3	73.87 (8)	Rb2—Fe2—Rb2^xxii^	60.0
O3^iii^—Rb2—O3	73.87 (7)	O1^vii^—Fe2—Rb2^xxiii^	146.11 (6)
O3^v^—Rb2—O1^vi^	80.68 (5)	O1^xiv^—Fe2—Rb2^xxiii^	58.77 (7)
O3^iii^—Rb2—O1^vi^	152.52 (6)	O1^xix^—Fe2—Rb2^xxiii^	70.91 (7)
O3—Rb2—O1^vi^	89.39 (6)	O1^v^—Fe2—Rb2^xxiii^	33.90 (6)
O3^v^—Rb2—O1^vii^	89.39 (6)	O1^xviii^—Fe2—Rb2^xxiii^	121.23 (7)
O3^iii^—Rb2—O1^vii^	80.68 (6)	O1^xvii^—Fe2—Rb2^xxiii^	109.09 (7)
O3—Rb2—O1^vii^	152.52 (6)	Rb2^xix^—Fe2—Rb2^xxiii^	120.0
O1^vi^—Rb2—O1^vii^	109.62 (3)	Rb2^xvii^—Fe2—Rb2^xxiii^	60.0
O3^v^—Rb2—O1^viii^	152.52 (6)	Rb2^xvi^—Fe2—Rb2^xxiii^	60.0
O3^iii^—Rb2—O1^viii^	89.39 (6)	Rb2—Fe2—Rb2^xxiii^	180.0
O3—Rb2—O1^viii^	80.68 (6)	Rb2^xxii^—Fe2—Rb2^xxiii^	120.0
O1^vi^—Rb2—O1^viii^	109.62 (3)	O1^xx^—As—O2	117.84 (10)
O1^vii^—Rb2—O1^viii^	109.62 (3)	O1^xx^—As—O4^ii^	107.24 (10)
O3^v^—Rb2—O4^ix^	113.60 (6)	O2—As—O4^ii^	114.38 (9)
O3^iii^—Rb2—O4^ix^	158.54 (6)	O1^xx^—As—O3	106.07 (11)
O3—Rb2—O4^ix^	127.10 (5)	O2—As—O3	104.17 (10)
O1^vi^—Rb2—O4^ix^	45.34 (4)	O4^ii^—As—O3	106.18 (9)
O1^vii^—Rb2—O4^ix^	79.36 (4)	O1^xx^—As—Rb2^xii^	63.12 (7)
O1^viii^—Rb2—O4^ix^	89.94 (5)	O2—As—Rb2^xii^	175.24 (7)
O3^v^—Rb2—O4^x^	158.54 (5)	O4^ii^—As—Rb2^xii^	68.86 (6)
O3^iii^—Rb2—O4^x^	127.10 (5)	O3—As—Rb2^xii^	71.28 (8)
O3—Rb2—O4^x^	113.60 (6)	O1^xx^—As—Rb1	140.53 (9)
O1^vi^—Rb2—O4^x^	79.36 (4)	O2—As—Rb1	57.00 (6)
O1^vii^—Rb2—O4^x^	89.94 (5)	O4^ii^—As—Rb1	109.61 (6)
O1^viii^—Rb2—O4^x^	45.34 (4)	O3—As—Rb1	49.92 (8)
O4^ix^—Rb2—O4^x^	45.37 (5)	Rb2^xii^—As—Rb1	119.004 (8)
O3^v^—Rb2—O4^xi^	127.10 (6)	O1^xx^—As—Rb2	77.51 (9)
O3^iii^—Rb2—O4^xi^	113.60 (6)	O2—As—Rb2	101.13 (6)
O3—Rb2—O4^xi^	158.54 (6)	O4^ii^—As—Rb2	134.56 (6)
O1^vi^—Rb2—O4^xi^	89.94 (5)	O3—As—Rb2	34.74 (7)
O1^vii^—Rb2—O4^xi^	45.34 (5)	Rb2^xii^—As—Rb2	74.367 (9)
O1^viii^—Rb2—O4^xi^	79.36 (4)	Rb1—As—Rb2	66.765 (14)
O4^ix^—Rb2—O4^xi^	45.37 (5)	O1^xx^—As—Rb2^xxiv^	46.08 (8)
O4^x^—Rb2—O4^xi^	45.37 (5)	O2—As—Rb2^xxiv^	125.75 (7)
O3^v^—Rb2—O3^xii^	122.48 (7)	O4^ii^—As—Rb2^xxiv^	63.02 (6)
O3^iii^—Rb2—O3^xii^	149.37 (7)	O3—As—Rb2^xxiv^	129.40 (8)
O3—Rb2—O3^xii^	85.69 (6)	Rb2^xii^—As—Rb2^xxiv^	58.581 (6)
O1^vi^—Rb2—O3^xii^	45.24 (5)	Rb1—As—Rb2^xxiv^	172.574 (7)
O1^vii^—Rb2—O3^xii^	121.79 (5)	Rb2—As—Rb2^xxiv^	117.235 (17)
O1^viii^—Rb2—O3^xii^	64.52 (5)	As^xxv^—O1—Fe2^xxvi^	140.90 (12)
O4^ix^—Rb2—O3^xii^	44.65 (4)	As^xxv^—O1—Rb2^vi^	91.09 (8)
O4^x^—Rb2—O3^xii^	42.56 (5)	Fe2^xxvi^—O1—Rb2^vi^	126.94 (8)
O4^xi^—Rb2—O3^xii^	78.68 (5)	As^xxv^—O1—Rb2^xxv^	79.81 (8)
O3^v^—Rb2—O3^xiii^	85.69 (6)	Fe2^xxvi^—O1—Rb2^xxv^	97.18 (8)
O3^iii^—Rb2—O3^xiii^	122.48 (8)	Rb2^vi^—O1—Rb2^xxv^	79.01 (4)
O3—Rb2—O3^xiii^	149.38 (7)	As^xxv^—O1—Rb2^xxvi^	118.94 (9)
O1^vi^—Rb2—O3^xiii^	64.52 (5)	Fe2^xxvi^—O1—Rb2^xxvi^	84.94 (7)
O1^vii^—Rb2—O3^xiii^	45.24 (5)	Rb2^vi^—O1—Rb2^xxvi^	73.02 (4)
O1^viii^—Rb2—O3^xiii^	121.79 (5)	Rb2^xxv^—O1—Rb2^xxvi^	146.08 (5)
O4^ix^—Rb2—O3^xiii^	42.56 (5)	As—O2—Fe1^xxiv^	122.52 (10)
O4^x^—Rb2—O3^xiii^	78.68 (5)	As—O2—Rb1	98.39 (7)
O4^xi^—Rb2—O3^xiii^	44.65 (4)	Fe1^xxiv^—O2—Rb1	128.56 (7)
O3^xii^—Rb2—O3^xiii^	86.44 (5)	As—O2—Rb2	59.04 (5)
O3^v^—Rb2—O3^xiv^	149.37 (7)	Fe1^xxiv^—O2—Rb2	163.04 (7)
O3^iii^—Rb2—O3^xiv^	85.69 (6)	Rb1—O2—Rb2	63.89 (3)
O3—Rb2—O3^xiv^	122.48 (8)	As—O3—Rb2	125.70 (10)
O1^vi^—Rb2—O3^xiv^	121.79 (5)	As—O3—Rb1	105.01 (9)
O1^vii^—Rb2—O3^xiv^	64.52 (5)	Rb2—O3—Rb1	95.22 (6)
O1^viii^—Rb2—O3^xiv^	45.24 (5)	As—O3—Rb2^xii^	81.77 (8)
O4^ix^—Rb2—O3^xiv^	78.68 (5)	Rb2—O3—Rb2^xii^	94.31 (6)
O4^x^—Rb2—O3^xiv^	44.65 (4)	Rb1—O3—Rb2^xii^	161.69 (7)
O4^xi^—Rb2—O3^xiv^	42.56 (4)	As—O3—H	107 (3)
O3^xii^—Rb2—O3^xiv^	86.44 (5)	Rb2—O3—H	122 (3)
O3^xiii^—Rb2—O3^xiv^	86.44 (5)	Rb1—O3—H	92 (3)
O3^v^—Rb2—As^xii^	105.92 (5)	Rb2^xii^—O3—H	69 (3)
O3^iii^—Rb2—As^xii^	175.05 (5)	As^ii^—O4—Fe1^xxvi^	129.96 (10)
O3—Rb2—As^xii^	101.27 (5)	As^ii^—O4—Rb2^xxvii^	85.04 (7)
O1^vi^—Rb2—As^xii^	25.79 (3)	Fe1^xxvi^—O4—Rb2^xxvii^	101.28 (7)
O1^vii^—Rb2—As^xii^	104.27 (4)	As^ii^—O4—Rb2^xxviii^	99.80 (7)
O1^viii^—Rb2—As^xii^	88.88 (4)	Fe1^xxvi^—O4—Rb2^xxviii^	128.35 (7)
O4^ix^—Rb2—As^xii^	26.10 (3)	Rb2^xxvii^—O4—Rb2^xxviii^	65.94 (3)
O4^x^—Rb2—As^xii^	53.64 (3)		

Symmetry codes: (i) *x*−*y*, −*y*, −*z*+3/2; (ii) −*x*, −*x*+*y*, −*z*+3/2; (iii) −*x*+*y*, −*x*, *z*; (iv) *y*, *x*, −*z*+3/2; (v) −*y*, *x*−*y*, *z*; (vi) −*x*+2/3, −*y*−2/3, −*z*+4/3; (vii) *y*+2/3, −*x*+*y*+4/3, −*z*+4/3; (viii) *x*−*y*−4/3, *x*−2/3, −*z*+4/3; (ix) *x*−1/3, *x*−*y*−2/3, *z*−1/6; (x) −*y*−1/3, −*x*+1/3, *z*−1/6; (xi) −*x*+*y*+2/3, *y*+1/3, *z*−1/6; (xii) −*x*−1/3, −*y*−2/3, −*z*+4/3; (xiii) *y*+2/3, −*x*+*y*+1/3, −*z*+4/3; (xiv) *x*−*y*−1/3, *x*+1/3, −*z*+4/3; (xv) −*y*, *x*−*y*+1, *z*; (xvi) *x*+1, *y*+1, *z*; (xvii) *x*, *y*+1, *z*; (xviii) −*x*+*y*+1, −*x*+1, *z*; (xix) −*x*+2/3, −*y*+1/3, −*z*+4/3; (xx) *x*−1, *y*, *z*; (xxi) −*y*+1/3, −*x*+2/3, *z*+1/6; (xxii) −*x*−1/3, −*y*+1/3, −*z*+4/3; (xxiii) −*x*+2/3, −*y*+4/3, −*z*+4/3; (xxiv) *x*−1, *y*−1, *z*; (xxv) *x*+1, *y*, *z*; (xxvi) *x*, *y*−1, *z*; (xxvii) −*y*+1/3, −*x*−1/3, *z*+1/6; (xxviii) *y*+1, *x*, −*z*+3/2.

### Rubidium iron bis[hydrogen arsenate(V)] (RbFeHAsO42) Hydrogen-bond geometry (Å, º)

**Table:**

*D*—H···*A*	*D*—H	H···*A*	*D*···*A*	*D*—H···*A*
O3—H···O4^xxix^	0.81 (3)	1.82 (3)	2.615 (3)	166 (4)

Symmetry code: (xxix) *y*, *x*−1, −*z*+3/2.

### Thallium iron bis[hydrogen arsenate(V)] (TlFeHAsO42) Crystal data

**Table:**

TlFe(HAsO_4_)_2_	*Z* = 3
*M**_r_* = 540.08	*F*(000) = 717
Triclinic, *P*1	*D*_x_ = 4.995 Mg m^−^^3^
*a* = 7.346 (2) Å	Mo *K*α radiation, λ = 0.71073 Å
*b* = 9.148 (2) Å	Cell parameters from 3867 reflections
*c* = 9.662 (2) Å	θ = 2.5–32.6°
α = 64.89 (3)°	µ = 33.58 mm^−^^1^
β = 70.51 (3)°	*T* = 293 K
γ = 69.94 (3)°	Short prismatic, colourless with red inclusions
*V* = 538.6 (3) Å^3^	0.10 × 0.05 × 0.04 mm

### Thallium iron bis[hydrogen arsenate(V)] (TlFeHAsO42) Data collection

**Table:**

Nonius KappaCCD single-crystal four-circle diffractometer	3391 reflections with *I* > 2σ(*I*)
Radiation source: fine-focus sealed tube	*R*_int_ = 0.021
φ and ω scans	θ_max_ = 32.6°, θ_min_ = 2.5°
Absorption correction: multi-scan (HKL SCALEPACK; Otwinowski *et al.*, 2003)	*h* = −11→11
*T*_min_ = 0.134, *T*_max_ = 0.347	*k* = −13→13
7723 measured reflections	*l* = −14→14
3906 independent reflections	

### Thallium iron bis[hydrogen arsenate(V)] (TlFeHAsO42) Refinement

**Table:**

Refinement on *F*^2^	Secondary atom site location: difference Fourier map
Least-squares matrix: full	Hydrogen site location: difference Fourier map
*R*[*F*^2^ > 2σ(*F*^2^)] = 0.022	Only H-atom displacement parameters refined
*wR*(*F*^2^) = 0.051	*w* = 1/[σ^2^(*F*_o_^2^) + (0.0193*P*)^2^ + 0.8062*P*] where *P* = (*F*_o_^2^ + 2*F*_c_^2^)/3
*S* = 1.06	(Δ/σ)_max_ = 0.005
3906 reflections	Δρ_max_ = 0.96 e Å^−^^3^
208 parameters	Δρ_min_ = −1.13 e Å^−^^3^
4 restraints	Extinction correction: SHELXL2016 (Sheldrick, 2015), Fc^*^=kFc[1+0.001xFc^2^λ^3^/sin(2θ)]^-1/4^
Primary atom site location: structure-invariant direct methods	Extinction coefficient: 0.0068 (2)

### Thallium iron bis[hydrogen arsenate(V)] (TlFeHAsO42) Special details

**Table:**

Geometry. All esds (except the esd in the dihedral angle between two l.s. planes) are estimated using the full covariance matrix. The cell esds are taken into account individually in the estimation of esds in distances, angles and torsion angles; correlations between esds in cell parameters are only used when they are defined by crystal symmetry. An approximate (isotropic) treatment of cell esds is used for estimating esds involving l.s. planes.

### Thallium iron bis[hydrogen arsenate(V)] (TlFeHAsO42) Fractional atomic coordinates and isotropic or equivalent isotropic displacement parameters (Å^2^)

**Table:**

	*x*	*y*	*z*	*U*_iso_*/*U*_eq_	Occ. (<1)
Tl1A	0.500000	1.000000	0.000000	0.0401 (16)	0.631 (3)
Tl1B	0.4695 (15)	0.9990 (11)	−0.0176 (13)	0.0299 (8)	0.1843 (14)
Tl2A	0.4052 (7)	0.8325 (4)	0.4667 (4)	0.0319 (6)	0.449 (3)
Tl2B	0.3531 (3)	0.8270 (4)	0.4840 (4)	0.0376 (3)	0.437 (3)
Tl2C	0.402 (3)	0.8510 (16)	0.4841 (15)	0.0286 (13)	0.114 (3)
Fe1	0.000000	0.500000	0.500000	0.00759 (10)	
Fe2	0.20590 (6)	0.72309 (5)	−0.05912 (5)	0.00754 (8)	
As1	0.45203 (4)	0.56033 (3)	0.21662 (3)	0.00716 (6)	
As2	−0.07841 (4)	0.87435 (3)	0.23460 (3)	0.00748 (6)	
As3	0.09158 (4)	0.35105 (3)	0.21621 (3)	0.00721 (6)	
O1	0.4101 (3)	0.7015 (2)	0.0441 (2)	0.0106 (4)	
O2	0.5906 (4)	0.6397 (3)	0.2680 (3)	0.0194 (5)	
O3	0.5713 (3)	0.3653 (2)	0.2221 (2)	0.0107 (4)	
O4	0.2440 (3)	0.5521 (3)	0.3531 (3)	0.0190 (5)	
O5	−0.0194 (3)	0.8151 (3)	0.0805 (2)	0.0130 (4)	
O6	−0.1590 (3)	0.7306 (2)	0.4022 (2)	0.0118 (4)	
O7	−0.2594 (3)	1.0506 (2)	0.2095 (2)	0.0108 (4)	
O8	0.1223 (3)	0.9190 (3)	0.2530 (3)	0.0173 (4)	
O9	−0.0703 (3)	0.4281 (3)	0.3527 (2)	0.0132 (4)	
O10	−0.0155 (3)	0.2336 (2)	0.1890 (2)	0.0114 (4)	
O11	0.1828 (3)	0.4911 (3)	0.0534 (2)	0.0135 (4)	
O12	0.2880 (3)	0.2007 (3)	0.2888 (3)	0.0159 (4)	
H2	0.694 (6)	0.570 (5)	0.295 (5)	0.033 (13)*	
H8	0.073100	1.037300	0.239694	0.08 (2)*	
H12	0.376 (7)	0.259 (6)	0.258 (6)	0.042 (14)*	

### Thallium iron bis[hydrogen arsenate(V)] (TlFeHAsO42) Atomic displacement parameters (Å^2^)

**Table:**

	*U*^11^	*U*^22^	*U*^33^	*U*^12^	*U*^13^	*U*^23^
Tl1A	0.060 (2)	0.0347 (7)	0.0312 (14)	−0.0317 (10)	−0.0034 (15)	−0.0060 (7)
Tl1B	0.0417 (9)	0.0227 (11)	0.0314 (14)	−0.0187 (13)	−0.0130 (8)	−0.0028 (9)
Tl2A	0.0396 (9)	0.0272 (9)	0.0211 (6)	0.0020 (7)	−0.0087 (6)	−0.0076 (4)
Tl2B	0.0433 (6)	0.0345 (5)	0.0234 (5)	−0.0040 (6)	−0.0136 (6)	0.0012 (3)
Tl2C	0.041 (4)	0.0191 (16)	0.019 (2)	0.0010 (14)	−0.010 (2)	−0.0056 (12)
Fe1	0.0087 (2)	0.0058 (2)	0.0072 (2)	−0.00065 (19)	−0.00228 (18)	−0.00170 (19)
Fe2	0.00853 (17)	0.00551 (16)	0.00764 (17)	−0.00077 (13)	−0.00218 (13)	−0.00183 (13)
As1	0.00724 (12)	0.00543 (12)	0.00788 (13)	−0.00077 (9)	−0.00187 (9)	−0.00189 (9)
As2	0.00859 (12)	0.00441 (12)	0.00832 (13)	−0.00028 (9)	−0.00242 (9)	−0.00177 (9)
As3	0.00828 (12)	0.00481 (12)	0.00847 (13)	−0.00135 (9)	−0.00238 (9)	−0.00200 (9)
O1	0.0117 (9)	0.0089 (9)	0.0088 (9)	−0.0023 (7)	−0.0047 (7)	0.0008 (7)
O2	0.0203 (11)	0.0144 (10)	0.0324 (13)	0.0006 (9)	−0.0162 (10)	−0.0123 (10)
O3	0.0101 (9)	0.0061 (8)	0.0130 (10)	0.0016 (7)	−0.0023 (7)	−0.0035 (7)
O4	0.0163 (10)	0.0165 (11)	0.0156 (11)	−0.0045 (9)	0.0068 (8)	−0.0051 (9)
O5	0.0140 (9)	0.0130 (10)	0.0124 (10)	−0.0009 (8)	−0.0019 (7)	−0.0074 (8)
O6	0.0138 (9)	0.0056 (8)	0.0092 (9)	−0.0004 (7)	−0.0024 (7)	0.0019 (7)
O7	0.0096 (9)	0.0060 (8)	0.0147 (10)	0.0008 (7)	−0.0038 (7)	−0.0028 (7)
O8	0.0149 (10)	0.0108 (10)	0.0307 (13)	−0.0015 (8)	−0.0125 (9)	−0.0070 (9)
O9	0.0114 (9)	0.0159 (10)	0.0132 (10)	0.0013 (8)	−0.0028 (7)	−0.0093 (8)
O10	0.0146 (9)	0.0081 (9)	0.0161 (10)	−0.0041 (7)	−0.0080 (8)	−0.0039 (8)
O11	0.0149 (10)	0.0080 (9)	0.0127 (10)	−0.0039 (8)	−0.0004 (7)	−0.0003 (8)
O12	0.0121 (10)	0.0119 (10)	0.0212 (11)	−0.0006 (8)	−0.0083 (8)	−0.0017 (9)

### Thallium iron bis[hydrogen arsenate(V)] (TlFeHAsO42) Geometric parameters (Å, º)

**Table:**

Tl1A—Tl1B^i^	0.327 (15)	Tl2B—O6^iii^	3.283 (3)
Tl1A—O1	2.853 (2)	Tl2B—O7^vii^	3.380 (6)
Tl1A—O1^i^	2.853 (2)	Tl2B—O4	3.650 (6)
Tl1A—O8^i^	3.094 (3)	Tl2B—As1^vi^	3.682 (3)
Tl1A—O8	3.094 (3)	Tl2B—O7^iii^	3.698 (3)
Tl1A—O2	3.227 (3)	Tl2B—O4^vi^	3.833 (4)
Tl1A—O2^i^	3.227 (3)	Tl2B—O12^vi^	3.843 (4)
Tl1A—O7^ii^	3.344 (2)	Tl2B—O2^vi^	3.861 (4)
Tl1A—O7^iii^	3.344 (2)	Tl2B—As3^viii^	3.879 (2)
Tl1A—O5^ii^	3.543 (2)	Tl2C—O3^vi^	2.711 (13)
Tl1A—O5^iii^	3.543 (2)	Tl2C—O12^v^	2.931 (13)
Tl1A—O12^iv^	3.615 (3)	Tl2C—O6^iii^	2.970 (18)
Tl1A—O12^v^	3.615 (3)	Tl2C—O2	3.111 (12)
Tl1A—As1^i^	3.7440 (14)	Tl2C—O7^vii^	3.197 (12)
Tl1A—As1	3.7440 (14)	Tl2C—O8	3.258 (14)
Tl1B—Tl1B^i^	0.65 (3)	Tl2C—O7^iii^	3.339 (17)
Tl1B—O1	2.687 (12)	Tl2C—O12^vi^	3.454 (14)
Tl1B—O8	3.014 (10)	Tl2C—O10^viii^	3.478 (17)
Tl1B—O7^ii^	3.022 (15)	Tl2C—O9^viii^	3.640 (16)
Tl1B—O1^i^	3.045 (14)	Tl2C—Tl2C^ix^	3.64 (3)
Tl1B—O2^i^	3.146 (8)	Tl2C—As1^vi^	3.706 (13)
Tl1B—O8^i^	3.205 (11)	Tl2C—O4^vi^	3.749 (14)
Tl1B—O5^ii^	3.286 (10)	Tl2C—As2^iii^	3.794 (18)
Tl1B—O2	3.338 (8)	Fe1—O4^viii^	1.942 (2)
Tl1B—O12^iv^	3.477 (12)	Fe1—O4	1.942 (2)
Tl1B—O7^iii^	3.666 (15)	Fe1—O6^viii^	2.015 (2)
Tl1B—As2^ii^	3.697 (13)	Fe1—O6	2.015 (2)
Tl1B—As1	3.701 (9)	Fe1—O9	2.060 (2)
Tl1B—O12^v^	3.775 (13)	Fe1—O9^viii^	2.060 (2)
Tl1B—O5^iii^	3.810 (11)	Fe2—O5	1.946 (2)
Tl2A—O3^vi^	2.804 (4)	Fe2—O11	1.970 (2)
Tl2A—O2	2.852 (4)	Fe2—O1	1.978 (2)
Tl2A—O6^iii^	2.936 (5)	Fe2—O10^x^	2.014 (2)
Tl2A—O12^v^	3.020 (4)	Fe2—O7^ii^	2.044 (2)
Tl2A—O8	3.091 (5)	Fe2—O3^iv^	2.065 (2)
Tl2A—O7^iii^	3.362 (5)	As1—O4	1.652 (2)
Tl2A—O7^vii^	3.450 (4)	As1—O1	1.668 (2)
Tl2A—O9^viii^	3.523 (5)	As1—O3	1.683 (2)
Tl2A—O10^viii^	3.572 (5)	As1—O2	1.720 (2)
Tl2A—O12^vi^	3.638 (5)	As2—O6	1.670 (2)
Tl2A—As1^vi^	3.688 (4)	As2—O5	1.671 (2)
Tl2A—O4^vi^	3.691 (4)	As2—O7	1.684 (2)
Tl2A—As2^iii^	3.763 (5)	As2—O8	1.738 (2)
Tl2A—O4	3.811 (5)	As3—O11	1.655 (2)
Tl2B—O3^vi^	2.758 (4)	As3—O10	1.6730 (19)
Tl2B—O8	2.919 (4)	As3—O9	1.679 (2)
Tl2B—O2	2.982 (6)	As3—O12	1.721 (2)
Tl2B—O12^v^	3.079 (4)	O2—H2	0.85 (3)
Tl2B—O9^viii^	3.204 (4)	O8—H8	0.982 (2)
Tl2B—O10^viii^	3.266 (4)	O12—H12	0.88 (3)
			
Tl1B^i^—Tl1A—O1	123.3 (15)	O5—As2—Tl1B	76.3 (2)
Tl1B^i^—Tl1A—O1^i^	56.7 (15)	O7—As2—Tl1B	108.47 (12)
O1—Tl1A—O1^i^	180.0	O8—As2—Tl1B	37.3 (2)
Tl1B^i^—Tl1A—O8^i^	72.9 (17)	Tl1B^ii^—As2—Tl1B	118.22 (18)
O1—Tl1A—O8^i^	115.26 (7)	Tl2A^xi^—As2—Tl1B	170.49 (16)
O1^i^—Tl1A—O8^i^	64.74 (7)	Tl2C^xi^—As2—Tl1B	166.8 (2)
Tl1B^i^—Tl1A—O8	107.1 (17)	Tl1A^xi^—As2—Tl1B	118.58 (16)
O1—Tl1A—O8	64.74 (7)	Tl2C^vii^—As2—Tl1B	112.2 (3)
O1^i^—Tl1A—O8	115.26 (7)	Tl2B^xi^—As2—Tl1B	170.96 (15)
O8^i^—Tl1A—O8	180.0	O6—As2—Tl2B^vii^	81.06 (8)
Tl1B^i^—Tl1A—O2	72.8 (15)	O5—As2—Tl2B^vii^	158.98 (8)
O1—Tl1A—O2	51.62 (6)	O7—As2—Tl2B^vii^	49.63 (8)
O1^i^—Tl1A—O2	128.38 (6)	O8—As2—Tl2B^vii^	77.26 (9)
O8^i^—Tl1A—O2	112.41 (7)	Tl1B^ii^—As2—Tl2B^vii^	99.31 (18)
O8—Tl1A—O2	67.59 (7)	Tl2A^xi^—As2—Tl2B^vii^	65.34 (6)
Tl1B^i^—Tl1A—O2^i^	107.2 (15)	Tl2C^xi^—As2—Tl2B^vii^	61.21 (17)
O1—Tl1A—O2^i^	128.39 (6)	Tl1A^xi^—As2—Tl2B^vii^	100.41 (4)
O1^i^—Tl1A—O2^i^	51.61 (6)	Tl2C^vii^—As2—Tl2B^vii^	6.4 (2)
O8^i^—Tl1A—O2^i^	67.59 (7)	Tl2B^xi^—As2—Tl2B^vii^	66.09 (10)
O8—Tl1A—O2^i^	112.41 (7)	Tl1B—As2—Tl2B^vii^	105.92 (19)
O2—Tl1A—O2^i^	180.0	O11—As3—O10	114.60 (11)
Tl1B^i^—Tl1A—O7^ii^	169.8 (17)	O11—As3—O9	114.77 (11)
O1—Tl1A—O7^ii^	52.66 (6)	O10—As3—O9	107.46 (11)
O1^i^—Tl1A—O7^ii^	127.34 (6)	O11—As3—O12	108.03 (11)
O8^i^—Tl1A—O7^ii^	99.91 (6)	O10—As3—O12	99.57 (10)
O8—Tl1A—O7^ii^	80.09 (6)	O9—As3—O12	111.41 (11)
O2—Tl1A—O7^ii^	104.24 (6)	O11—As3—Tl2B^viii^	151.04 (8)
O2^i^—Tl1A—O7^ii^	75.76 (6)	O10—As3—Tl2B^viii^	56.44 (11)
Tl1B^i^—Tl1A—O7^iii^	10.2 (17)	O9—As3—Tl2B^viii^	54.30 (11)
O1—Tl1A—O7^iii^	127.34 (6)	O12—As3—Tl2B^viii^	100.81 (9)
O1^i^—Tl1A—O7^iii^	52.66 (6)	O11—As3—Tl2A^viii^	149.26 (9)
O8^i^—Tl1A—O7^iii^	80.09 (6)	O10—As3—Tl2A^viii^	55.87 (9)
O8—Tl1A—O7^iii^	99.91 (6)	O9—As3—Tl2A^viii^	54.20 (9)
O2—Tl1A—O7^iii^	75.76 (6)	O12—As3—Tl2A^viii^	102.56 (9)
O2^i^—Tl1A—O7^iii^	104.24 (6)	Tl2B^viii^—As3—Tl2A^viii^	1.80 (6)
O7^ii^—Tl1A—O7^iii^	180.0	O11—As3—Tl2C^viii^	148.9 (2)
Tl1B^i^—Tl1A—O5^ii^	143.2 (16)	O10—As3—Tl2C^viii^	52.14 (17)
O1—Tl1A—O5^ii^	83.29 (6)	O9—As3—Tl2C^viii^	57.83 (17)
O1^i^—Tl1A—O5^ii^	96.71 (6)	O12—As3—Tl2C^viii^	102.3 (2)
O8^i^—Tl1A—O5^ii^	121.76 (6)	Tl2B^viii^—As3—Tl2C^viii^	4.5 (2)
O8—Tl1A—O5^ii^	58.24 (6)	Tl2A^viii^—As3—Tl2C^viii^	3.75 (14)
O2—Tl1A—O5^ii^	120.71 (6)	O11—As3—Tl1B^iv^	84.07 (15)
O2^i^—Tl1A—O5^ii^	59.29 (6)	O10—As3—Tl1B^iv^	69.2 (2)
O7^ii^—Tl1A—O5^ii^	46.78 (5)	O9—As3—Tl1B^iv^	159.27 (14)
O7^iii^—Tl1A—O5^ii^	133.22 (5)	O12—As3—Tl1B^iv^	51.33 (18)
Tl1B^i^—Tl1A—O5^iii^	36.8 (16)	Tl2B^viii^—As3—Tl1B^iv^	113.00 (17)
O1—Tl1A—O5^iii^	96.71 (6)	Tl2A^viii^—As3—Tl1B^iv^	113.69 (16)
O1^i^—Tl1A—O5^iii^	83.29 (6)	Tl2C^viii^—As3—Tl1B^iv^	110.6 (2)
O8^i^—Tl1A—O5^iii^	58.24 (6)	O11—As3—Tl1A^xiii^	84.12 (8)
O8—Tl1A—O5^iii^	121.76 (6)	O10—As3—Tl1A^xiii^	65.45 (7)
O2—Tl1A—O5^iii^	59.29 (6)	O9—As3—Tl1A^xiii^	160.59 (8)
O2^i^—Tl1A—O5^iii^	120.71 (6)	O12—As3—Tl1A^xiii^	55.16 (8)
O7^ii^—Tl1A—O5^iii^	133.22 (5)	Tl2B^viii^—As3—Tl1A^xiii^	111.13 (8)
O7^iii^—Tl1A—O5^iii^	46.78 (5)	Tl2A^viii^—As3—Tl1A^xiii^	111.69 (6)
O5^ii^—Tl1A—O5^iii^	180.00 (3)	Tl2C^viii^—As3—Tl1A^xiii^	108.43 (16)
Tl1B^i^—Tl1A—O12^iv^	117.2 (17)	Tl1B^iv^—As3—Tl1A^xiii^	4.35 (19)
O1—Tl1A—O12^iv^	57.83 (6)	O11—As3—Tl1B^xiii^	84.20 (14)
O1^i^—Tl1A—O12^iv^	122.17 (6)	O10—As3—Tl1B^xiii^	61.76 (19)
O8^i^—Tl1A—O12^iv^	59.93 (6)	O9—As3—Tl1B^xiii^	161.02 (14)
O8—Tl1A—O12^iv^	120.07 (6)	O12—As3—Tl1B^xiii^	58.96 (17)
O2—Tl1A—O12^iv^	88.55 (6)	Tl2B^viii^—As3—Tl1B^xiii^	109.19 (17)
O2^i^—Tl1A—O12^iv^	91.45 (6)	Tl2A^viii^—As3—Tl1B^xiii^	109.63 (16)
O7^ii^—Tl1A—O12^iv^	52.68 (5)	Tl2C^viii^—As3—Tl1B^xiii^	106.3 (2)
O7^iii^—Tl1A—O12^iv^	127.32 (5)	Tl1B^iv^—As3—Tl1B^xiii^	8.6 (4)
O5^ii^—Tl1A—O12^iv^	98.17 (5)	Tl1A^xiii^—As3—Tl1B^xiii^	4.27 (18)
O5^iii^—Tl1A—O12^iv^	81.83 (5)	O11—As3—Tl2C^xiii^	129.0 (2)
Tl1B^i^—Tl1A—O12^v^	62.8 (17)	O10—As3—Tl2C^xiii^	80.0 (2)
O1—Tl1A—O12^v^	122.17 (6)	O9—As3—Tl2C^xiii^	104.96 (19)
O1^i^—Tl1A—O12^v^	57.83 (6)	O12—As3—Tl2C^xiii^	23.7 (2)
O8^i^—Tl1A—O12^v^	120.07 (6)	Tl2B^viii^—As3—Tl2C^xiii^	78.9 (2)
O8—Tl1A—O12^v^	59.93 (6)	Tl2A^viii^—As3—Tl2C^xiii^	80.5 (2)
O2—Tl1A—O12^v^	91.45 (6)	Tl2C^viii^—As3—Tl2C^xiii^	79.8 (4)
O2^i^—Tl1A—O12^v^	88.55 (6)	Tl1B^iv^—As3—Tl2C^xiii^	54.5 (2)
O7^ii^—Tl1A—O12^v^	127.32 (5)	Tl1A^xiii^—As3—Tl2C^xiii^	56.81 (16)
O7^iii^—Tl1A—O12^v^	52.68 (5)	Tl1B^xiii^—As3—Tl2C^xiii^	59.2 (2)
O5^ii^—Tl1A—O12^v^	81.83 (5)	O11—As3—Tl2B^xiii^	132.75 (8)
O5^iii^—Tl1A—O12^v^	98.17 (5)	O10—As3—Tl2B^xiii^	74.02 (9)
O12^iv^—Tl1A—O12^v^	180.00 (6)	O9—As3—Tl2B^xiii^	104.89 (10)
Tl1B^i^—Tl1A—As1^i^	79.9 (15)	O12—As3—Tl2B^xiii^	29.66 (8)
O1—Tl1A—As1^i^	155.08 (4)	Tl2B^viii^—As3—Tl2B^xiii^	74.28 (6)
O1^i^—Tl1A—As1^i^	24.92 (4)	Tl2A^viii^—As3—Tl2B^xiii^	75.87 (6)
O8^i^—Tl1A—As1^i^	59.16 (5)	Tl2C^viii^—As3—Tl2B^xiii^	74.87 (19)
O8—Tl1A—As1^i^	120.84 (5)	Tl1B^iv^—As3—Tl2B^xiii^	54.38 (13)
O2—Tl1A—As1^i^	152.70 (4)	Tl1A^xiii^—As3—Tl2B^xiii^	56.21 (6)
O2^i^—Tl1A—As1^i^	27.30 (4)	Tl1B^xiii^—As3—Tl2B^xiii^	58.18 (13)
O7^ii^—Tl1A—As1^i^	102.87 (4)	Tl2C^xiii^—As3—Tl2B^xiii^	6.2 (2)
O7^iii^—Tl1A—As1^i^	77.13 (4)	As1—O1—Fe2	126.13 (11)
O5^ii^—Tl1A—As1^i^	81.04 (5)	As1—O1—Tl1B	114.3 (3)
O5^iii^—Tl1A—As1^i^	98.96 (5)	Fe2—O1—Tl1B	111.4 (3)
O12^iv^—Tl1A—As1^i^	105.49 (5)	As1—O1—Tl1A	108.97 (9)
O12^v^—Tl1A—As1^i^	74.51 (5)	Fe2—O1—Tl1A	117.26 (9)
Tl1B^i^—Tl1A—As1	100.1 (15)	Tl1B—O1—Tl1A	5.8 (2)
O1—Tl1A—As1	24.92 (4)	As1—O1—Tl1B^i^	104.2 (2)
O1^i^—Tl1A—As1	155.08 (4)	Fe2—O1—Tl1B^i^	122.4 (2)
O8^i^—Tl1A—As1	120.84 (5)	Tl1B—O1—Tl1B^i^	11.0 (4)
O8—Tl1A—As1	59.16 (5)	Tl1A—O1—Tl1B^i^	5.16 (19)
O2—Tl1A—As1	27.30 (4)	As1—O1—Tl2A	55.90 (7)
O2^i^—Tl1A—As1	152.70 (4)	Fe2—O1—Tl2A	135.14 (10)
O7^ii^—Tl1A—As1	77.13 (4)	Tl1B—O1—Tl2A	61.8 (3)
O7^iii^—Tl1A—As1	102.87 (4)	Tl1A—O1—Tl2A	57.72 (6)
O5^ii^—Tl1A—As1	98.96 (5)	Tl1B^i^—O1—Tl2A	54.3 (2)
O5^iii^—Tl1A—As1	81.04 (5)	As1—O1—Tl2B	55.35 (7)
O12^iv^—Tl1A—As1	74.51 (5)	Fe2—O1—Tl2B	131.07 (8)
O12^v^—Tl1A—As1	105.49 (5)	Tl1B—O1—Tl2B	64.2 (3)
As1^i^—Tl1A—As1	180.0	Tl1A—O1—Tl2B	60.43 (5)
Tl1B^i^—Tl1B—O1	117.4 (14)	Tl1B^i^—O1—Tl2B	57.3 (2)
Tl1B^i^—Tl1B—O8	101.1 (18)	Tl2A—O1—Tl2B	4.56 (6)
O1—Tl1B—O8	67.8 (3)	As1—O1—Tl2C	56.97 (16)
Tl1B^i^—Tl1B—O7^ii^	168.7 (18)	Fe2—O1—Tl2C	134.8 (2)
O1—Tl1B—O7^ii^	58.0 (3)	Tl1B—O1—Tl2C	60.8 (3)
O8—Tl1B—O7^ii^	86.8 (3)	Tl1A—O1—Tl2C	56.75 (17)
Tl1B^i^—Tl1B—O1^i^	51.6 (15)	Tl1B^i^—O1—Tl2C	53.4 (3)
O1—Tl1B—O1^i^	169.0 (4)	Tl2A—O1—Tl2C	1.07 (18)
O8—Tl1B—O1^i^	112.0 (4)	Tl2B—O1—Tl2C	4.9 (2)
O7^ii^—Tl1B—O1^i^	132.7 (3)	As1—O1—Tl2C^xii^	150.86 (17)
Tl1B^i^—Tl1B—O2^i^	101.5 (16)	Fe2—O1—Tl2C^xii^	47.1 (2)
O1—Tl1B—O2^i^	139.6 (5)	Tl1B—O1—Tl2C^xii^	91.3 (3)
O8—Tl1B—O2^i^	117.0 (2)	Tl1A—O1—Tl2C^xii^	95.80 (16)
O7^ii^—Tl1B—O2^i^	81.7 (3)	Tl1B^i^—O1—Tl2C^xii^	99.8 (3)
O1^i^—Tl1B—O2^i^	51.03 (17)	Tl2A—O1—Tl2C^xii^	152.55 (13)
Tl1B^i^—Tl1B—O8^i^	67.3 (17)	Tl2B—O1—Tl2C^xii^	153.67 (13)
O1—Tl1B—O8^i^	116.7 (3)	Tl2C—O1—Tl2C^xii^	151.5 (3)
O8—Tl1B—O8^i^	168.4 (5)	As1—O1—Tl2A^i^	151.74 (10)
O7^ii^—Tl1B—O8^i^	104.7 (4)	Fe2—O1—Tl2A^i^	81.86 (8)
O1^i^—Tl1B—O8^i^	61.3 (2)	Tl1B—O1—Tl2A^i^	47.4 (3)
O2^i^—Tl1B—O8^i^	67.3 (2)	Tl1A—O1—Tl2A^i^	51.24 (6)
Tl1B^i^—Tl1B—O5^ii^	139.8 (18)	Tl1B^i^—O1—Tl2A^i^	54.8 (2)
O1—Tl1B—O5^ii^	91.0 (4)	Tl2A—O1—Tl2A^i^	108.96 (6)
O8—Tl1B—O5^ii^	62.0 (2)	Tl2B—O1—Tl2A^i^	111.56 (8)
O7^ii^—Tl1B—O5^ii^	51.3 (2)	Tl2C—O1—Tl2A^i^	107.99 (18)
O1^i^—Tl1B—O5^ii^	98.6 (2)	Tl2C^xii^—O1—Tl2A^i^	45.12 (17)
O2^i^—Tl1B—O5^ii^	62.93 (16)	As1—O1—Tl2C^i^	153.1 (2)
O8^i^—Tl1B—O5^ii^	126.7 (3)	Fe2—O1—Tl2C^i^	80.01 (19)
Tl1B^i^—Tl1B—O2	67.4 (14)	Tl1B—O1—Tl2C^i^	50.5 (3)
O1—Tl1B—O2	51.18 (14)	Tl1A—O1—Tl2C^i^	54.32 (13)
O8—Tl1B—O2	67.03 (19)	Tl1B^i^—O1—Tl2C^i^	57.9 (3)
O7^ii^—Tl1B—O2	109.2 (4)	Tl2A—O1—Tl2C^i^	112.04 (15)
O1^i^—Tl1B—O2	118.1 (4)	Tl2B—O1—Tl2C^i^	114.66 (15)
O2^i^—Tl1B—O2	168.9 (5)	Tl2C—O1—Tl2C^i^	111.07 (16)
O8^i^—Tl1B—O2	106.8 (2)	Tl2C^xii^—O1—Tl2C^i^	42.2 (3)
O5^ii^—Tl1B—O2	125.3 (3)	Tl2A^i^—O1—Tl2C^i^	3.11 (12)
Tl1B^i^—Tl1B—O12^iv^	112.4 (17)	As1—O2—Tl2A	120.80 (15)
O1—Tl1B—O12^iv^	60.9 (3)	As1—O2—Tl2B	114.06 (13)
O8—Tl1B—O12^iv^	127.2 (4)	Tl2A—O2—Tl2B	6.91 (12)
O7^ii^—Tl1B—O12^iv^	56.4 (2)	As1—O2—Tl2C	122.7 (3)
O1^i^—Tl1B—O12^iv^	120.7 (3)	Tl2A—O2—Tl2C	1.9 (4)
O2^i^—Tl1B—O12^iv^	95.5 (3)	Tl2B—O2—Tl2C	8.8 (3)
O8^i^—Tl1B—O12^iv^	60.63 (19)	As1—O2—Tl1B^i^	99.0 (3)
O5^ii^—Tl1B—O12^iv^	106.2 (4)	Tl2A—O2—Tl1B^i^	79.2 (2)
O2—Tl1B—O12^iv^	89.1 (2)	Tl2B—O2—Tl1B^i^	81.3 (2)
Tl1B^i^—Tl1B—O7^iii^	9.3 (16)	Tl2C—O2—Tl1B^i^	79.2 (3)
O1—Tl1B—O7^iii^	121.3 (3)	As1—O2—Tl1A	93.34 (9)
O8—Tl1B—O7^iii^	94.6 (3)	Tl2A—O2—Tl1A	81.85 (9)
O7^ii^—Tl1B—O7^iii^	178.0 (3)	Tl2B—O2—Tl1A	83.31 (8)
O1^i^—Tl1B—O7^iii^	47.9 (2)	Tl2C—O2—Tl1A	81.9 (2)
O2^i^—Tl1B—O7^iii^	98.9 (4)	Tl1B^i^—O2—Tl1A	5.7 (3)
O8^i^—Tl1B—O7^iii^	73.9 (3)	As1—O2—Tl1B	88.0 (3)
O5^ii^—Tl1B—O7^iii^	130.7 (3)	Tl2A—O2—Tl1B	84.3 (2)
O2—Tl1B—O7^iii^	70.2 (2)	Tl2B—O2—Tl1B	85.2 (2)
O12^iv^—Tl1B—O7^iii^	121.6 (3)	Tl2C—O2—Tl1B	84.6 (3)
Tl1B^i^—Tl1B—As2^ii^	162.4 (16)	Tl1B^i^—O2—Tl1B	11.1 (5)
O1—Tl1B—As2^ii^	79.5 (4)	Tl1A—O2—Tl1B	5.4 (3)
O8—Tl1B—As2^ii^	80.1 (3)	As1—O2—Tl2B^vi^	71.06 (10)
O7^ii^—Tl1B—As2^ii^	26.69 (11)	Tl2A—O2—Tl2B^vi^	109.95 (13)
O1^i^—Tl1B—As2^ii^	111.5 (2)	Tl2B—O2—Tl2B^vi^	107.08 (11)
O2^i^—Tl1B—As2^ii^	63.25 (18)	Tl2C—O2—Tl2B^vi^	110.3 (3)
O8^i^—Tl1B—As2^ii^	110.9 (3)	Tl1B^i^—O2—Tl2B^vi^	168.9 (3)
O5^ii^—Tl1B—As2^ii^	26.86 (11)	Tl1A—O2—Tl2B^vi^	163.67 (10)
O2—Tl1B—As2^ii^	127.7 (4)	Tl1B—O2—Tl2B^vi^	158.5 (3)
O12^iv^—Tl1B—As2^ii^	79.4 (3)	As1—O2—Tl2A^vi^	67.86 (10)
O7^iii^—Tl1B—As2^ii^	155.0 (3)	Tl2A—O2—Tl2A^vi^	107.71 (11)
Tl1B^i^—Tl1B—As1	95.1 (14)	Tl2B—O2—Tl2A^vi^	104.32 (10)
O1—Tl1B—As1	24.26 (9)	Tl2C—O2—Tl2A^vi^	108.2 (3)
O8—Tl1B—As1	60.26 (18)	Tl1B^i^—O2—Tl2A^vi^	166.9 (3)
O7^ii^—Tl1B—As1	81.8 (3)	Tl1A—O2—Tl2A^vi^	161.19 (9)
O1^i^—Tl1B—As1	145.5 (4)	Tl1B—O2—Tl2A^vi^	155.8 (3)
O2^i^—Tl1B—As1	163.4 (5)	Tl2B^vi^—O2—Tl2A^vi^	5.22 (6)
O8^i^—Tl1B—As1	119.0 (2)	As1—O2—Tl2C^vi^	65.1 (2)
O5^ii^—Tl1B—As1	104.8 (3)	Tl2A—O2—Tl2C^vi^	111.0 (2)
O2—Tl1B—As1	27.68 (8)	Tl2B—O2—Tl2C^vi^	107.5 (2)
O12^iv^—Tl1B—As1	76.7 (2)	Tl2C—O2—Tl2C^vi^	111.5 (2)
O7^iii^—Tl1B—As1	97.7 (2)	Tl1B^i^—O2—Tl2C^vi^	163.8 (3)
As2^ii^—Tl1B—As1	100.6 (3)	Tl1A—O2—Tl2C^vi^	158.2 (2)
Tl1B^i^—Tl1B—O12^v^	58.4 (17)	Tl1B—O2—Tl2C^vi^	152.9 (3)
O1—Tl1B—O12^v^	121.9 (3)	Tl2B^vi^—O2—Tl2C^vi^	6.2 (2)
O8—Tl1B—O12^v^	58.5 (2)	Tl2A^vi^—O2—Tl2C^vi^	3.62 (15)
O7^ii^—Tl1B—O12^v^	132.8 (3)	As1—O2—H2	113 (3)
O1^i^—Tl1B—O12^v^	54.7 (2)	Tl2A—O2—H2	111 (3)
O2^i^—Tl1B—O12^v^	87.0 (3)	Tl2B—O2—H2	114 (3)
O8^i^—Tl1B—O12^v^	112.6 (4)	Tl2C—O2—H2	109 (3)
O5^ii^—Tl1B—O12^v^	82.9 (2)	Tl1B^i^—O2—H2	131 (3)
O2—Tl1B—O12^v^	87.0 (3)	Tl1A—O2—H2	135 (3)
O12^iv^—Tl1B—O12^v^	170.8 (4)	Tl1B—O2—H2	139 (3)
O7^iii^—Tl1B—O12^v^	49.2 (2)	Tl2B^vi^—O2—H2	52 (3)
As2^ii^—Tl1B—O12^v^	109.6 (2)	Tl2A^vi^—O2—H2	58 (3)
As1—Tl1B—O12^v^	103.2 (3)	Tl2C^vi^—O2—H2	58 (3)
Tl1B^i^—Tl1B—O5^iii^	33.9 (14)	As1—O3—Fe2^iv^	131.25 (12)
O1—Tl1B—O5^iii^	93.69 (19)	As1—O3—Tl2C^vi^	112.9 (3)
O8—Tl1B—O5^iii^	116.0 (3)	Fe2^iv^—O3—Tl2C^vi^	110.1 (3)
O7^ii^—Tl1B—O5^iii^	135.1 (3)	As1—O3—Tl2B^vi^	109.61 (14)
O1^i^—Tl1B—O5^iii^	76.4 (3)	Fe2^iv^—O3—Tl2B^vi^	108.65 (11)
O2^i^—Tl1B—O5^iii^	115.3 (3)	Tl2C^vi^—O3—Tl2B^vi^	10.1 (4)
O8^i^—Tl1B—O5^iii^	54.44 (17)	As1—O3—Tl2A^vi^	107.96 (12)
O5^ii^—Tl1B—O5^iii^	173.6 (5)	Fe2^iv^—O3—Tl2A^vi^	113.66 (11)
O2—Tl1B—O5^iii^	55.60 (15)	Tl2C^vi^—O3—Tl2A^vi^	5.4 (2)
O12^iv^—Tl1B—O5^iii^	79.93 (17)	Tl2B^vi^—O3—Tl2A^vi^	7.66 (10)
O7^iii^—Tl1B—O5^iii^	42.95 (15)	As1—O3—Tl1B^iv^	135.47 (14)
As2^ii^—Tl1B—O5^iii^	159.0 (4)	Fe2^iv^—O3—Tl1B^iv^	55.10 (13)
As1—Tl1B—O5^iii^	78.19 (14)	Tl2C^vi^—O3—Tl1B^iv^	98.4 (3)
O12^v^—Tl1B—O5^iii^	91.0 (3)	Tl2B^vi^—O3—Tl1B^iv^	106.05 (16)
O3^vi^—Tl2A—O2	110.62 (13)	Tl2A^vi^—O3—Tl1B^iv^	103.81 (15)
O3^vi^—Tl2A—O6^iii^	84.49 (12)	As1—O3—Tl2C^xiii^	134.1 (2)
O2—Tl2A—O6^iii^	63.62 (10)	Fe2^iv^—O3—Tl2C^xiii^	91.18 (19)
O3^vi^—Tl2A—O12^v^	135.34 (15)	Tl2C^vi^—O3—Tl2C^xiii^	51.0 (4)
O2—Tl2A—O12^v^	113.29 (14)	Tl2B^vi^—O3—Tl2C^xiii^	60.46 (16)
O6^iii^—Tl2A—O12^v^	107.85 (16)	Tl2A^vi^—O3—Tl2C^xiii^	55.88 (18)
O3^vi^—Tl2A—O8	136.34 (18)	Tl1B^iv^—O3—Tl2C^xiii^	50.50 (18)
O2—Tl2A—O8	72.45 (11)	As1—O3—Tl2A^xiii^	135.71 (10)
O6^iii^—Tl2A—O8	129.21 (14)	Fe2^iv^—O3—Tl2A^xiii^	88.82 (8)
O12^v^—Tl2A—O8	67.16 (10)	Tl2C^vi^—O3—Tl2A^xiii^	53.5 (3)
O3^vi^—Tl2A—O7^iii^	124.09 (17)	Tl2B^vi^—O3—Tl2A^xiii^	62.88 (9)
O2—Tl2A—O7^iii^	80.54 (12)	Tl2A^vi^—O3—Tl2A^xiii^	58.48 (13)
O6^iii^—Tl2A—O7^iii^	50.58 (9)	Tl1B^iv^—O3—Tl2A^xiii^	47.62 (12)
O12^v^—Tl2A—O7^iii^	57.75 (9)	Tl2C^xiii^—O3—Tl2A^xiii^	2.89 (14)
O8—Tl2A—O7^iii^	99.55 (11)	As1—O3—Tl1A^xiii^	132.86 (9)
O3^vi^—Tl2A—O7^vii^	51.02 (8)	Fe2^iv^—O3—Tl1A^xiii^	56.68 (5)
O2—Tl2A—O7^vii^	161.15 (13)	Tl2C^vi^—O3—Tl1A^xiii^	100.0 (3)
O6^iii^—Tl2A—O7^vii^	106.12 (14)	Tl2B^vi^—O3—Tl1A^xiii^	107.82 (12)
O12^v^—Tl2A—O7^vii^	84.49 (11)	Tl2A^vi^—O3—Tl1A^xiii^	105.31 (10)
O8—Tl2A—O7^vii^	122.69 (13)	Tl1B^iv^—O3—Tl1A^xiii^	2.65 (11)
O7^iii^—Tl2A—O7^vii^	105.65 (13)	Tl2C^xiii^—O3—Tl1A^xiii^	51.31 (14)
O3^vi^—Tl2A—O9^viii^	67.53 (10)	Tl2A^xiii^—O3—Tl1A^xiii^	48.45 (5)
O2—Tl2A—O9^viii^	84.95 (13)	As1—O3—Tl2B^xiii^	132.28 (9)
O6^iii^—Tl2A—O9^viii^	127.21 (12)	Fe2^iv^—O3—Tl2B^xiii^	91.64 (8)
O12^v^—Tl2A—O9^viii^	123.73 (15)	Tl2C^vi^—O3—Tl2B^xiii^	54.3 (3)
O8—Tl2A—O9^viii^	69.50 (12)	Tl2B^vi^—O3—Tl2B^xiii^	63.88 (13)
O7^iii^—Tl2A—O9^viii^	164.03 (13)	Tl2A^vi^—O3—Tl2B^xiii^	59.06 (9)
O7^vii^—Tl2A—O9^viii^	90.22 (11)	Tl1B^iv^—O3—Tl2B^xiii^	48.44 (13)
O3^vi^—Tl2A—O10^viii^	50.72 (8)	Tl2C^xiii^—O3—Tl2B^xiii^	3.8 (2)
O2—Tl2A—O10^viii^	129.09 (16)	Tl2A^xiii^—O3—Tl2B^xiii^	3.54 (5)
O6^iii^—Tl2A—O10^viii^	135.10 (13)	Tl1A^xiii^—O3—Tl2B^xiii^	49.07 (6)
O12^v^—Tl2A—O10^viii^	103.61 (12)	As1—O3—Tl1B	26.48 (12)
O8—Tl2A—O10^viii^	92.25 (13)	Fe2^iv^—O3—Tl1B	105.07 (14)
O7^iii^—Tl2A—O10^viii^	150.35 (13)	Tl2C^vi^—O3—Tl1B	131.6 (3)
O7^vii^—Tl2A—O10^viii^	46.17 (7)	Tl2B^vi^—O3—Tl1B	124.7 (2)
O9^viii^—Tl2A—O10^viii^	44.78 (7)	Tl2A^vi^—O3—Tl1B	126.15 (18)
O3^vi^—Tl2A—O12^vi^	48.01 (9)	Tl1B^iv^—O3—Tl1B	129.18 (12)
O2—Tl2A—O12^vi^	114.65 (14)	Tl2C^xiii^—O3—Tl1B	158.3 (2)
O6^iii^—Tl2A—O12^vi^	54.68 (9)	Tl2A^xiii^—O3—Tl1B	158.75 (14)
O12^v^—Tl2A—O12^vi^	104.24 (13)	Tl1A^xiii^—O3—Tl1B	127.28 (17)
O8—Tl2A—O12^vi^	171.01 (13)	Tl2B^xiii^—O3—Tl1B	155.26 (13)
O7^iii^—Tl2A—O12^vi^	76.97 (12)	As1—O3—Tl1B^xiii^	130.50 (12)
O7^vii^—Tl2A—O12^vi^	51.73 (8)	Fe2^iv^—O3—Tl1B^xiii^	58.15 (12)
O9^viii^—Tl2A—O12^vi^	115.55 (11)	Tl2C^vi^—O3—Tl1B^xiii^	101.3 (3)
O10^viii^—Tl2A—O12^vi^	87.20 (9)	Tl2B^vi^—O3—Tl1B^xiii^	109.41 (15)
O3^vi^—Tl2A—As1^vi^	25.72 (5)	Tl2A^vi^—O3—Tl1B^xiii^	106.65 (14)
O2—Tl2A—As1^vi^	84.90 (10)	Tl1B^iv^—O3—Tl1B^xiii^	5.04 (19)
O6^iii^—Tl2A—As1^vi^	72.19 (9)	Tl2C^xiii^—O3—Tl1B^xiii^	52.12 (17)
O12^v^—Tl2A—As1^vi^	160.16 (14)	Tl2A^xiii^—O3—Tl1B^xiii^	49.29 (11)
O8—Tl2A—As1^vi^	128.92 (15)	Tl1A^xiii^—O3—Tl1B^xiii^	2.40 (9)
O7^iii^—Tl2A—As1^vi^	121.50 (13)	Tl2B^xiii^—O3—Tl1B^xiii^	49.72 (12)
O7^vii^—Tl2A—As1^vi^	76.68 (8)	Tl1B—O3—Tl1B^xiii^	125.5 (2)
O9^viii^—Tl2A—As1^vi^	63.26 (8)	As1—O4—Fe1	166.48 (15)
O10^viii^—Tl2A—As1^vi^	67.47 (8)	As1—O4—Tl2B	89.90 (10)
O12^vi^—Tl2A—As1^vi^	58.90 (7)	Fe1—O4—Tl2B	103.33 (9)
O3^vi^—Tl2A—O4^vi^	46.68 (8)	As1—O4—Tl2A^vi^	76.96 (11)
O2—Tl2A—O4^vi^	68.41 (9)	Fe1—O4—Tl2A^vi^	98.20 (11)
O6^iii^—Tl2A—O4^vi^	47.49 (8)	Tl2B—O4—Tl2A^vi^	97.68 (9)
O12^v^—Tl2A—O4^vi^	153.38 (18)	As1—O4—Tl2C^vi^	75.8 (3)
O8—Tl2A—O4^vi^	133.41 (14)	Fe1—O4—Tl2C^vi^	98.5 (3)
O7^iii^—Tl2A—O4^vi^	97.99 (12)	Tl2B—O4—Tl2C^vi^	101.6 (2)
O7^vii^—Tl2A—O4^vi^	92.92 (10)	Tl2A^vi^—O4—Tl2C^vi^	4.18 (17)
O9^viii^—Tl2A—O4^vi^	82.69 (9)	As1—O4—Tl2A	84.81 (11)
O10^viii^—Tl2A—O4^vi^	93.26 (10)	Fe1—O4—Tl2A	108.35 (11)
O12^vi^—Tl2A—O4^vi^	55.57 (8)	Tl2B—O4—Tl2A	5.20 (7)
As1^vi^—Tl2A—O4^vi^	25.87 (4)	Tl2A^vi^—O4—Tl2A	95.45 (10)
O3^vi^—Tl2A—As2^iii^	107.80 (14)	Tl2C^vi^—O4—Tl2A	99.3 (2)
O2—Tl2A—As2^iii^	64.63 (10)	As1—O4—Tl2B^vi^	72.21 (9)
O6^iii^—Tl2A—As2^iii^	25.22 (6)	Fe1—O4—Tl2B^vi^	103.35 (9)
O12^v^—Tl2A—As2^iii^	84.29 (12)	Tl2B—O4—Tl2B^vi^	95.21 (11)
O8—Tl2A—As2^iii^	111.92 (11)	Tl2A^vi^—O4—Tl2B^vi^	5.28 (8)
O7^iii^—Tl2A—As2^iii^	26.59 (5)	Tl2C^vi^—O4—Tl2B^vi^	7.2 (3)
O7^vii^—Tl2A—As2^iii^	113.46 (13)	Tl2A—O4—Tl2B^vi^	92.62 (11)
O9^viii^—Tl2A—As2^iii^	145.94 (11)	As1—O4—Tl2C	86.4 (3)
O10^viii^—Tl2A—As2^iii^	155.68 (13)	Fe1—O4—Tl2C	106.8 (2)
O12^vi^—Tl2A—As2^iii^	68.51 (9)	Tl2B—O4—Tl2C	3.7 (3)
As1^vi^—Tl2A—As2^iii^	97.41 (10)	Tl2A^vi^—O4—Tl2C	95.8 (2)
O4^vi^—Tl2A—As2^iii^	72.43 (9)	Tl2C^vi^—O4—Tl2C	99.6 (2)
O3^vi^—Tl2A—O4	100.56 (13)	Tl2A—O4—Tl2C	1.6 (3)
O2—Tl2A—O4	44.83 (9)	Tl2B^vi^—O4—Tl2C	93.1 (2)
O6^iii^—Tl2A—O4	105.38 (11)	As1—O4—Tl1B	50.23 (13)
O12^v^—Tl2A—O4	116.01 (13)	Fe1—O4—Tl1B	139.29 (14)
O8—Tl2A—O4	49.45 (9)	Tl2B—O4—Tl1B	62.09 (19)
O7^iii^—Tl2A—O4	120.12 (11)	Tl2A^vi^—O4—Tl1B	120.48 (15)
O7^vii^—Tl2A—O4	134.10 (14)	Tl2C^vi^—O4—Tl1B	121.0 (3)
O9^viii^—Tl2A—O4	43.94 (7)	Tl2A—O4—Tl1B	59.2 (2)
O10^viii^—Tl2A—O4	88.11 (12)	Tl2B^vi^—O4—Tl1B	115.20 (13)
O12^vi^—Tl2A—O4	139.43 (11)	Tl2C—O4—Tl1B	60.4 (3)
As1^vi^—Tl2A—O4	82.21 (9)	As1—O4—Tl1A	49.70 (7)
O4^vi^—Tl2A—O4	84.55 (10)	Fe1—O4—Tl1A	140.71 (10)
As2^iii^—Tl2A—O4	109.33 (10)	Tl2B—O4—Tl1A	58.81 (5)
O3^vi^—Tl2A—Tl1B^i^	161.45 (17)	Tl2A^vi^—O4—Tl1A	117.68 (9)
O2—Tl2A—Tl1B^i^	53.76 (17)	Tl2C^vi^—O4—Tl1A	118.5 (3)
O6^iii^—Tl2A—Tl1B^i^	79.28 (18)	Tl2A—O4—Tl1A	55.77 (6)
O12^v^—Tl2A—Tl1B^i^	59.60 (16)	Tl2B^vi^—O4—Tl1A	112.41 (7)
O8—Tl2A—Tl1B^i^	53.9 (2)	Tl2C—O4—Tl1A	56.96 (19)
O7^iii^—Tl2A—Tl1B^i^	49.1 (2)	Tl1B—O4—Tl1A	4.04 (18)
O7^vii^—Tl2A—Tl1B^i^	143.0 (2)	As1—O4—Tl1B^i^	49.42 (13)
O9^viii^—Tl2A—Tl1B^i^	116.1 (2)	Fe1—O4—Tl1B^i^	141.78 (14)
O10^viii^—Tl2A—Tl1B^i^	145.3 (2)	Tl2B—O4—Tl1B^i^	55.70 (18)
O12^vi^—Tl2A—Tl1B^i^	124.9 (2)	Tl2A^vi^—O4—Tl1B^i^	114.90 (14)
As1^vi^—Tl2A—Tl1B^i^	137.33 (16)	Tl2C^vi^—O4—Tl1B^i^	115.9 (3)
O4^vi^—Tl2A—Tl1B^i^	114.80 (15)	Tl2A—O4—Tl1B^i^	52.46 (19)
As2^iii^—Tl2A—Tl1B^i^	58.25 (19)	Tl2B^vi^—O4—Tl1B^i^	109.66 (13)
O4—Tl2A—Tl1B^i^	75.5 (2)	Tl2C—O4—Tl1B^i^	53.7 (3)
O3^vi^—Tl2B—O8	149.22 (12)	Tl1B—O4—Tl1B^i^	7.9 (3)
O3^vi^—Tl2B—O2	108.16 (12)	Tl1A—O4—Tl1B^i^	3.91 (17)
O8—Tl2B—O2	73.17 (13)	As2—O5—Fe2	142.35 (13)
O3^vi^—Tl2B—O12^v^	134.7 (2)	As2—O5—Tl1B^ii^	90.4 (3)
O8—Tl2B—O12^v^	68.56 (8)	Fe2—O5—Tl1B^ii^	126.1 (2)
O2—Tl2B—O12^v^	108.07 (11)	As2—O5—Tl1A^xi^	93.59 (9)
O3^vi^—Tl2B—O9^viii^	73.06 (8)	Fe2—O5—Tl1A^xi^	123.14 (9)
O8—Tl2B—O9^viii^	76.24 (10)	Tl1B^ii^—O5—Tl1A^xi^	3.4 (2)
O2—Tl2B—O9^viii^	88.85 (14)	As2—O5—Tl1B^xi^	96.3 (2)
O12^v^—Tl2B—O9^viii^	133.58 (9)	Fe2—O5—Tl1B^xi^	120.6 (2)
O3^vi^—Tl2B—O10^viii^	54.99 (8)	Tl1B^ii^—O5—Tl1B^xi^	6.4 (5)
O8—Tl2B—O10^viii^	102.08 (8)	Tl1A^xi^—O5—Tl1B^xi^	3.0 (2)
O2—Tl2B—O10^viii^	136.68 (14)	As2—O5—Tl1B	80.61 (19)
O12^v^—Tl2B—O10^viii^	109.78 (14)	Fe2—O5—Tl1B	68.44 (17)
O9^viii^—Tl2B—O10^viii^	49.37 (6)	Tl1B^ii^—O5—Tl1B	131.69 (18)
O3^vi^—Tl2B—O6^iii^	78.90 (8)	Tl1A^xi^—O5—Tl1B	133.51 (11)
O8—Tl2B—O6^iii^	122.70 (14)	Tl1B^xi^—O5—Tl1B	135.0 (2)
O2—Tl2B—O6^iii^	58.07 (8)	As2—O5—Tl1A	78.54 (8)
O12^v^—Tl2B—O6^iii^	98.30 (9)	Fe2—O5—Tl1A	69.75 (6)
O9^viii^—Tl2B—O6^iii^	126.44 (12)	Tl1B^ii^—O5—Tl1A	133.1 (2)
O10^viii^—Tl2B—O6^iii^	133.58 (11)	Tl1A^xi^—O5—Tl1A	135.05 (6)
O3^vi^—Tl2B—O7^vii^	52.16 (10)	Tl1B^xi^—O5—Tl1A	136.64 (17)
O8—Tl2B—O7^vii^	131.34 (12)	Tl1B—O5—Tl1A	2.70 (16)
O2—Tl2B—O7^vii^	155.48 (9)	As2—O5—Tl2A^xi^	49.78 (8)
O12^v^—Tl2B—O7^vii^	84.81 (13)	Fe2—O5—Tl2A^xi^	157.69 (10)
O9^viii^—Tl2B—O7^vii^	97.20 (9)	Tl1B^ii^—O5—Tl2A^xi^	54.9 (2)
O10^viii^—Tl2B—O7^vii^	48.95 (8)	Tl1A^xi^—O5—Tl2A^xi^	56.74 (6)
O6^iii^—Tl2B—O7^vii^	100.23 (12)	Tl1B^xi^—O5—Tl2A^xi^	58.40 (17)
O3^vi^—Tl2B—O4	105.48 (12)	Tl1B—O5—Tl2A^xi^	129.96 (19)
O8—Tl2B—O4	52.13 (10)	Tl1A—O5—Tl2A^xi^	128.08 (7)
O2—Tl2B—O4	46.54 (9)	As2—O5—Tl1B^i^	76.70 (17)
O12^v^—Tl2B—O4	119.12 (12)	Fe2—O5—Tl1B^i^	70.94 (16)
O9^viii^—Tl2B—O4	46.87 (8)	Tl1B^ii^—O5—Tl1B^i^	134.2 (3)
O10^viii^—Tl2B—O4	95.76 (9)	Tl1A^xi^—O5—Tl1B^i^	136.33 (10)
O6^iii^—Tl2B—O4	101.98 (13)	Tl1B^xi^—O5—Tl1B^i^	138.05 (15)
O7^vii^—Tl2B—O4	144.05 (8)	Tl1B—O5—Tl1B^i^	5.1 (3)
O3^vi^—Tl2B—As1^vi^	25.50 (5)	Tl1A—O5—Tl1B^i^	2.40 (13)
O8—Tl2B—As1^vi^	135.81 (15)	Tl2A^xi^—O5—Tl1B^i^	126.37 (17)
O2—Tl2B—As1^vi^	83.25 (10)	As2—O5—Tl2C^xi^	46.81 (17)
O12^v^—Tl2B—As1^vi^	155.60 (15)	Fe2—O5—Tl2C^xi^	160.19 (18)
O9^viii^—Tl2B—As1^vi^	66.26 (6)	Tl1B^ii^—O5—Tl2C^xi^	56.2 (3)
O10^viii^—Tl2B—As1^vi^	70.68 (6)	Tl1A^xi^—O5—Tl2C^xi^	58.14 (17)
O6^iii^—Tl2B—As1^vi^	68.82 (6)	Tl1B^xi^—O5—Tl2C^xi^	59.9 (2)
O7^vii^—Tl2B—As1^vi^	77.62 (8)	Tl1B—O5—Tl2C^xi^	126.9 (2)
O4—Tl2B—As1^vi^	84.53 (9)	Tl1A—O5—Tl2C^xi^	125.04 (17)
O3^vi^—Tl2B—O7^iii^	114.48 (10)	Tl2A^xi^—O5—Tl2C^xi^	3.08 (18)
O8—Tl2B—O7^iii^	95.62 (9)	Tl1B^i^—O5—Tl2C^xi^	123.3 (2)
O2—Tl2B—O7^iii^	73.45 (8)	As2—O5—Tl2B^xi^	51.91 (8)
O12^v^—Tl2B—O7^iii^	53.44 (7)	Fe2—O5—Tl2B^xi^	156.29 (10)
O9^viii^—Tl2B—O7^iii^	162.11 (18)	Tl1B^ii^—O5—Tl2B^xi^	53.55 (19)
O10^viii^—Tl2B—O7^iii^	148.46 (17)	Tl1A^xi^—O5—Tl2B^xi^	55.28 (5)
O6^iii^—Tl2B—O7^iii^	45.34 (6)	Tl1B^xi^—O5—Tl2B^xi^	56.85 (17)
O7^vii^—Tl2B—O7^iii^	100.05 (12)	Tl1B—O5—Tl2B^xi^	132.05 (19)
O4—Tl2B—O7^iii^	115.66 (12)	Tl1A—O5—Tl2B^xi^	130.19 (7)
As1^vi^—Tl2B—O7^iii^	113.00 (6)	Tl2A^xi^—O5—Tl2B^xi^	2.14 (10)
O3^vi^—Tl2B—O4^vi^	44.76 (7)	Tl1B^i^—O5—Tl2B^xi^	128.49 (17)
O8—Tl2B—O4^vi^	134.38 (18)	Tl2C^xi^—O5—Tl2B^xi^	5.15 (19)
O2—Tl2B—O4^vi^	65.24 (9)	As2—O6—Fe1	126.05 (12)
O12^v^—Tl2B—O4^vi^	141.63 (9)	As2—O6—Tl2A^xi^	106.26 (12)
O9^viii^—Tl2B—O4^vi^	84.79 (8)	Fe1—O6—Tl2A^xi^	124.82 (12)
O10^viii^—Tl2B—O4^vi^	95.78 (8)	As2—O6—Tl2C^xi^	106.3 (3)
O6^iii^—Tl2B—O4^vi^	44.76 (6)	Fe1—O6—Tl2C^xi^	126.3 (3)
O7^vii^—Tl2B—O4^vi^	91.56 (9)	Tl2A^xi^—O6—Tl2C^xi^	5.3 (2)
O4—Tl2B—O4^vi^	84.79 (11)	As2—O6—Tl2B^xi^	108.64 (10)
As1^vi^—Tl2B—O4^vi^	25.29 (4)	Fe1—O6—Tl2B^xi^	122.23 (10)
O7^iii^—Tl2B—O4^vi^	90.02 (7)	Tl2A^xi^—O6—Tl2B^xi^	2.60 (10)
O3^vi^—Tl2B—O12^vi^	45.24 (8)	Tl2C^xi^—O6—Tl2B^xi^	6.8 (3)
O8—Tl2B—O12^vi^	165.47 (10)	As2—O6—Tl2C^vii^	78.7 (2)
O2—Tl2B—O12^vi^	106.12 (8)	Fe1—O6—Tl2C^vii^	135.73 (19)
O12^v^—Tl2B—O12^vi^	98.54 (12)	Tl2A^xi^—O6—Tl2C^vii^	64.8 (2)
O9^viii^—Tl2B—O12^vi^	118.27 (9)	Tl2C^xi^—O6—Tl2C^vii^	59.8 (4)
O10^viii^—Tl2B—O12^vi^	88.37 (11)	Tl2B^xi^—O6—Tl2C^vii^	66.6 (3)
O6^iii^—Tl2B—O12^vi^	50.52 (7)	As2—O6—Tl2B^vii^	76.31 (8)
O7^vii^—Tl2B—O12^vi^	50.28 (8)	Fe1—O6—Tl2B^vii^	133.00 (9)
O4—Tl2B—O12^vi^	137.64 (10)	Tl2A^xi^—O6—Tl2B^vii^	70.73 (9)
As1^vi^—Tl2B—O12^vi^	57.16 (6)	Tl2C^xi^—O6—Tl2B^vii^	65.8 (2)
O7^iii^—Tl2B—O12^vi^	70.64 (7)	Tl2B^xi^—O6—Tl2B^vii^	72.57 (11)
O4^vi^—Tl2B—O12^vi^	52.86 (6)	Tl2C^vii^—O6—Tl2B^vii^	6.0 (3)
O3^vi^—Tl2B—O2^vi^	46.10 (7)	As2—O6—Tl2A^vii^	79.49 (9)
O8—Tl2B—O2^vi^	109.73 (14)	Fe1—O6—Tl2A^vii^	133.98 (9)
O2—Tl2B—O2^vi^	72.92 (11)	Tl2A^xi^—O6—Tl2A^vii^	65.97 (14)
O12^v^—Tl2B—O2^vi^	177.42 (8)	Tl2C^xi^—O6—Tl2A^vii^	61.0 (2)
O9^viii^—Tl2B—O2^vi^	43.85 (7)	Tl2B^xi^—O6—Tl2A^vii^	67.76 (10)
O10^viii^—Tl2B—O2^vi^	68.46 (7)	Tl2C^vii^—O6—Tl2A^vii^	1.8 (2)
O6^iii^—Tl2B—O2^vi^	84.25 (9)	Tl2B^vii^—O6—Tl2A^vii^	4.97 (7)
O7^vii^—Tl2B—O2^vi^	95.15 (8)	As2—O6—Tl1B^ii^	55.67 (16)
O4—Tl2B—O2^vi^	59.71 (8)	Fe1—O6—Tl1B^ii^	138.50 (18)
As1^vi^—Tl2B—O2^vi^	26.23 (4)	Tl2A^xi^—O6—Tl1B^ii^	59.44 (15)
O7^iii^—Tl2B—O2^vi^	129.04 (9)	Tl2C^xi^—O6—Tl1B^ii^	62.4 (3)
O4^vi^—Tl2B—O2^vi^	40.95 (6)	Tl2B^xi^—O6—Tl1B^ii^	60.96 (14)
O12^vi^—Tl2B—O2^vi^	83.38 (7)	Tl2C^vii^—O6—Tl1B^ii^	85.3 (2)
O3^vi^—Tl2B—As3^viii^	66.57 (6)	Tl2B^vii^—O6—Tl1B^ii^	88.46 (17)
O8—Tl2B—As3^viii^	84.53 (8)	Tl2A^vii^—O6—Tl1B^ii^	87.14 (17)
O2—Tl2B—As3^viii^	114.04 (13)	As2—O6—Tl2B	75.09 (8)
O12^v^—Tl2B—As3^viii^	119.79 (9)	Fe1—O6—Tl2B	76.97 (8)
O9^viii^—Tl2B—As3^viii^	25.19 (4)	Tl2A^xi^—O6—Tl2B	139.02 (8)
O10^viii^—Tl2B—As3^viii^	25.27 (4)	Tl2C^xi^—O6—Tl2B	133.7 (2)
O6^iii^—Tl2B—As3^viii^	140.22 (8)	Tl2B^xi^—O6—Tl2B	140.17 (16)
O7^vii^—Tl2B—As3^viii^	74.20 (7)	Tl2C^vii^—O6—Tl2B	75.8 (3)
O4—Tl2B—As3^viii^	70.61 (7)	Tl2B^vii^—O6—Tl2B	69.97 (6)
As1^vi^—Tl2B—As3^viii^	71.55 (5)	Tl2A^vii^—O6—Tl2B	74.36 (11)
O7^iii^—Tl2B—As3^viii^	172.07 (14)	Tl1B^ii^—O6—Tl2B	129.88 (17)
O4^vi^—Tl2B—As3^viii^	95.54 (7)	As2—O6—Tl1A^xi^	57.71 (7)
O12^vi^—Tl2B—As3^viii^	108.40 (9)	Fe1—O6—Tl1A^xi^	136.87 (8)
O2^vi^—Tl2B—As3^viii^	57.80 (6)	Tl2A^xi^—O6—Tl1A^xi^	58.46 (8)
O3^vi^—Tl2C—O12^v^	145.5 (4)	Tl2C^xi^—O6—Tl1A^xi^	61.6 (2)
O3^vi^—Tl2C—O6^iii^	85.5 (4)	Tl2B^xi^—O6—Tl1A^xi^	59.90 (7)
O12^v^—Tl2C—O6^iii^	109.3 (5)	Tl2C^vii^—O6—Tl1A^xi^	86.74 (19)
O3^vi^—Tl2C—O2	105.8 (4)	Tl2B^vii^—O6—Tl1A^xi^	90.01 (6)
O12^v^—Tl2C—O2	108.6 (4)	Tl2A^vii^—O6—Tl1A^xi^	88.54 (7)
O6^iii^—Tl2C—O2	60.2 (3)	Tl1B^ii^—O6—Tl1A^xi^	2.28 (17)
O3^vi^—Tl2C—O7^vii^	54.9 (2)	Tl2B—O6—Tl1A^xi^	132.08 (6)
O12^v^—Tl2C—O7^vii^	90.7 (3)	As2—O6—Tl2A	73.27 (9)
O6^iii^—Tl2C—O7^vii^	112.0 (4)	Fe1—O6—Tl2A	76.91 (8)
O2—Tl2C—O7^vii^	160.6 (5)	Tl2A^xi^—O6—Tl2A	141.05 (13)
O3^vi^—Tl2C—O8	132.8 (6)	Tl2C^xi^—O6—Tl2A	135.7 (3)
O12^v^—Tl2C—O8	66.0 (3)	Tl2B^xi^—O6—Tl2A	142.30 (9)
O6^iii^—Tl2C—O8	121.8 (4)	Tl2C^vii^—O6—Tl2A	77.4 (2)
O2—Tl2C—O8	67.0 (2)	Tl2B^vii^—O6—Tl2A	71.48 (7)
O7^vii^—Tl2C—O8	125.7 (5)	Tl2A^vii^—O6—Tl2A	75.96 (9)
O3^vi^—Tl2C—O7^iii^	128.4 (6)	Tl1B^ii^—O6—Tl2A	128.38 (17)
O12^v^—Tl2C—O7^iii^	58.8 (3)	Tl2B—O6—Tl2A	2.59 (8)
O6^iii^—Tl2C—O7^iii^	50.6 (3)	Tl1A^xi^—O6—Tl2A	130.56 (6)
O2—Tl2C—O7^iii^	77.4 (3)	As2—O7—Fe2^ii^	122.44 (11)
O7^vii^—Tl2C—O7^iii^	112.3 (4)	As2—O7—Tl1B^ii^	99.60 (17)
O8—Tl2C—O7^iii^	96.7 (3)	Fe2^ii^—O7—Tl1B^ii^	97.94 (18)
O3^vi^—Tl2C—O12^vi^	50.8 (2)	As2—O7—Tl2C^vii^	110.0 (3)
O12^v^—Tl2C—O12^vi^	111.0 (4)	Fe2^ii^—O7—Tl2C^vii^	94.7 (3)
O6^iii^—Tl2C—O12^vi^	56.8 (3)	Tl1B^ii^—O7—Tl2C^vii^	134.3 (4)
O2—Tl2C—O12^vi^	113.1 (5)	As2—O7—Tl2C^xi^	92.1 (3)
O7^vii^—Tl2C—O12^vi^	55.3 (2)	Fe2^ii^—O7—Tl2C^xi^	145.3 (3)
O8—Tl2C—O12^vi^	176.4 (5)	Tl1B^ii^—O7—Tl2C^xi^	77.4 (3)
O7^iii^—Tl2C—O12^vi^	79.9 (4)	Tl2C^vii^—O7—Tl2C^xi^	67.7 (4)
O3^vi^—Tl2C—O10^viii^	52.4 (3)	As2—O7—Tl1A^xi^	100.56 (9)
O12^v^—Tl2C—O10^viii^	107.9 (5)	Fe2^ii^—O7—Tl1A^xi^	97.82 (8)
O6^iii^—Tl2C—O10^viii^	137.8 (4)	Tl1B^ii^—O7—Tl1A^xi^	1.10 (17)
O2—Tl2C—O10^viii^	123.5 (5)	Tl2C^vii^—O7—Tl1A^xi^	133.2 (3)
O7^vii^—Tl2C—O10^viii^	48.5 (2)	Tl2C^xi^—O7—Tl1A^xi^	76.9 (2)
O8—Tl2C—O10^viii^	91.2 (4)	As2—O7—Tl2A^xi^	90.08 (10)
O7^iii^—Tl2C—O10^viii^	159.0 (4)	Fe2^ii^—O7—Tl2A^xi^	147.47 (11)
O12^vi^—Tl2C—O10^viii^	91.7 (3)	Tl1B^ii^—O7—Tl2A^xi^	73.5 (2)
O3^vi^—Tl2C—O9^viii^	66.5 (3)	Tl2C^vii^—O7—Tl2A^xi^	72.4 (3)
O12^v^—Tl2C—O9^viii^	122.7 (5)	Tl2C^xi^—O7—Tl2A^xi^	4.73 (18)
O6^iii^—Tl2C—O9^viii^	122.0 (4)	Tl1A^xi^—O7—Tl2A^xi^	73.06 (8)
O2—Tl2C—O9^viii^	79.4 (3)	As2—O7—Tl2B^vii^	108.05 (10)
O7^vii^—Tl2C—O9^viii^	92.3 (4)	Fe2^ii^—O7—Tl2B^vii^	89.65 (8)
O8—Tl2C—O9^viii^	66.3 (3)	Tl1B^ii^—O7—Tl2B^vii^	141.32 (19)
O7^iii^—Tl2C—O9^viii^	155.4 (4)	Tl2C^vii^—O7—Tl2B^vii^	7.8 (3)
O12^vi^—Tl2C—O9^viii^	117.3 (4)	Tl2C^xi^—O7—Tl2B^vii^	75.10 (18)
O10^viii^—Tl2C—O9^viii^	44.6 (2)	Tl1A^xi^—O7—Tl2B^vii^	140.26 (7)
O3^vi^—Tl2C—Tl2C^ix^	93.7 (5)	Tl2A^xi^—O7—Tl2B^vii^	79.80 (8)
O12^v^—Tl2C—Tl2C^ix^	62.3 (4)	As2—O7—Tl2A^vii^	110.54 (11)
O6^iii^—Tl2C—Tl2C^ix^	75.3 (6)	Fe2^ii^—O7—Tl2A^vii^	92.84 (10)
O2—Tl2C—Tl2C^ix^	128.9 (8)	Tl1B^ii^—O7—Tl2A^vii^	135.4 (2)
O7^vii^—Tl2C—Tl2C^ix^	58.0 (3)	Tl2C^vii^—O7—Tl2A^vii^	2.0 (3)
O8—Tl2C—Tl2C^ix^	128.2 (6)	Tl2C^xi^—O7—Tl2A^vii^	69.6 (2)
O7^iii^—Tl2C—Tl2C^ix^	54.3 (4)	Tl1A^xi^—O7—Tl2A^vii^	134.39 (10)
O12^vi^—Tl2C—Tl2C^ix^	48.7 (3)	Tl2A^xi^—O7—Tl2A^vii^	74.35 (13)
O10^viii^—Tl2C—Tl2C^ix^	105.9 (5)	Tl2B^vii^—O7—Tl2A^vii^	6.18 (9)
O9^viii^—Tl2C—Tl2C^ix^	150.3 (6)	As2—O7—Tl1B^xi^	101.35 (15)
O3^vi^—Tl2C—As1^vi^	24.72 (13)	Fe2^ii^—O7—Tl1B^xi^	97.73 (15)
O12^v^—Tl2C—As1^vi^	169.5 (4)	Tl1B^ii^—O7—Tl1B^xi^	2.0 (3)
O6^iii^—Tl2C—As1^vi^	71.6 (3)	Tl2C^vii^—O7—Tl1B^xi^	132.4 (4)
O2—Tl2C—As1^vi^	81.2 (3)	Tl2C^xi^—O7—Tl1B^xi^	76.5 (3)
O7^vii^—Tl2C—As1^vi^	79.5 (3)	Tl1A^xi^—O7—Tl1B^xi^	0.90 (14)
O8—Tl2C—As1^vi^	122.9 (4)	Tl2A^xi^—O7—Tl1B^xi^	72.67 (17)
O7^iii^—Tl2C—As1^vi^	121.7 (5)	Tl2B^vii^—O7—Tl1B^xi^	139.38 (16)
O12^vi^—Tl2C—As1^vi^	60.3 (2)	Tl2A^vii^—O7—Tl1B^xi^	133.53 (18)
O10^viii^—Tl2C—As1^vi^	68.2 (3)	As2—O7—Tl2B^xi^	92.59 (10)
O9^viii^—Tl2C—As1^vi^	62.0 (2)	Fe2^ii^—O7—Tl2B^xi^	144.92 (10)
Tl2C^ix^—Tl2C—As1^vi^	108.7 (5)	Tl1B^ii^—O7—Tl2B^xi^	72.2 (2)
O3^vi^—Tl2C—O4^vi^	46.0 (2)	Tl2C^vii^—O7—Tl2B^xi^	72.5 (3)
O12^v^—Tl2C—O4^vi^	155.8 (6)	Tl2C^xi^—O7—Tl2B^xi^	5.3 (2)
O6^iii^—Tl2C—O4^vi^	46.7 (2)	Tl1A^xi^—O7—Tl2B^xi^	71.63 (8)
O2—Tl2C—O4^vi^	65.4 (3)	Tl2A^xi^—O7—Tl2B^xi^	2.70 (7)
O7^vii^—Tl2C—O4^vi^	96.1 (3)	Tl2B^vii^—O7—Tl2B^xi^	79.95 (12)
O8—Tl2C—O4^vi^	125.5 (3)	Tl2A^vii^—O7—Tl2B^xi^	74.37 (10)
O7^iii^—Tl2C—O4^vi^	97.3 (4)	Tl1B^xi^—O7—Tl2B^xi^	71.21 (17)
O12^vi^—Tl2C—O4^vi^	56.5 (2)	As2—O7—Tl1B	52.90 (12)
O10^viii^—Tl2C—O4^vi^	93.8 (3)	Fe2^ii^—O7—Tl1B	69.77 (11)
O9^viii^—Tl2C—O4^vi^	80.4 (3)	Tl1B^ii^—O7—Tl1B	113.2 (2)
Tl2C^ix^—Tl2C—O4^vi^	102.1 (6)	Tl2C^vii^—O7—Tl1B	112.4 (4)
As1^vi^—Tl2C—O4^vi^	25.60 (10)	Tl2C^xi^—O7—Tl1B	143.9 (3)
O3^vi^—Tl2C—As2^iii^	109.1 (5)	Tl1A^xi^—O7—Tl1B	114.21 (18)
O12^v^—Tl2C—As2^iii^	84.9 (4)	Tl2A^xi^—O7—Tl1B	142.66 (12)
O6^iii^—Tl2C—As2^iii^	25.01 (15)	Tl2B^vii^—O7—Tl1B	105.03 (18)
O2—Tl2C—As2^iii^	62.3 (3)	Tl2A^vii^—O7—Tl1B	111.1 (2)
O7^vii^—Tl2C—As2^iii^	119.1 (4)	Tl1B^xi^—O7—Tl1B	115.0 (3)
O8—Tl2C—As2^iii^	107.4 (3)	Tl2B^xi^—O7—Tl1B	145.27 (11)
O7^iii^—Tl2C—As2^iii^	26.34 (14)	As2—O8—Tl2B	140.00 (13)
O12^vi^—Tl2C—As2^iii^	70.0 (3)	As2—O8—Tl1B	122.2 (3)
O10^viii^—Tl2C—As2^iii^	160.7 (4)	Tl2B—O8—Tl1B	92.5 (3)
O9^viii^—Tl2C—As2^iii^	139.2 (4)	As2—O8—Tl2A	145.64 (14)
Tl2C^ix^—Tl2C—As2^iii^	66.7 (5)	Tl2B—O8—Tl2A	6.34 (9)
As1^vi^—Tl2C—As2^iii^	96.6 (4)	Tl1B—O8—Tl2A	86.2 (3)
O4^vi^—Tl2C—As2^iii^	71.5 (3)	As2—O8—Tl1A	128.10 (11)
O3^vi^—Tl2C—Tl2A^ix^	92.3 (3)	Tl2B—O8—Tl1A	86.74 (7)
O12^v^—Tl2C—Tl2A^ix^	62.8 (2)	Tl1B—O8—Tl1A	6.0 (3)
O6^iii^—Tl2C—Tl2A^ix^	77.0 (3)	Tl2A—O8—Tl1A	80.39 (10)
O2—Tl2C—Tl2A^ix^	131.1 (5)	As2—O8—Tl1B^i^	133.6 (3)
O7^vii^—Tl2C—Tl2A^ix^	55.8 (2)	Tl2B—O8—Tl1B^i^	81.3 (2)
O8—Tl2C—Tl2A^ix^	128.8 (3)	Tl1B—O8—Tl1B^i^	11.6 (5)
O7^iii^—Tl2C—Tl2A^ix^	56.5 (2)	Tl2A—O8—Tl1B^i^	74.9 (3)
O12^vi^—Tl2C—Tl2A^ix^	48.24 (18)	Tl1A—O8—Tl1B^i^	5.6 (3)
O10^viii^—Tl2C—Tl2A^ix^	103.7 (3)	As2—O8—Tl2C	146.3 (3)
O9^viii^—Tl2C—Tl2A^ix^	148.1 (4)	Tl2B—O8—Tl2C	6.4 (3)
Tl2C^ix^—Tl2C—Tl2A^ix^	2.3 (3)	Tl1B—O8—Tl2C	87.6 (4)
As1^vi^—Tl2C—Tl2A^ix^	108.0 (3)	Tl2A—O8—Tl2C	4.0 (2)
O4^vi^—Tl2C—Tl2A^ix^	102.4 (3)	Tl1A—O8—Tl2C	81.7 (3)
As2^iii^—Tl2C—Tl2A^ix^	68.9 (2)	Tl1B^i^—O8—Tl2C	76.2 (4)
O4^viii^—Fe1—O4	180.0	As2—O8—Tl2B^vii^	78.89 (10)
O4^viii^—Fe1—O6^viii^	91.70 (10)	Tl2B—O8—Tl2B^vii^	88.66 (8)
O4—Fe1—O6^viii^	88.30 (10)	Tl1B—O8—Tl2B^vii^	137.2 (2)
O4^viii^—Fe1—O6	88.30 (10)	Tl2A—O8—Tl2B^vii^	93.12 (12)
O4—Fe1—O6	91.70 (10)	Tl1A—O8—Tl2B^vii^	135.59 (8)
O6^viii^—Fe1—O6	180.0	Tl1B^i^—O8—Tl2B^vii^	133.6 (2)
O4^viii^—Fe1—O9	87.04 (9)	Tl2C—O8—Tl2B^vii^	89.6 (3)
O4—Fe1—O9	92.96 (9)	As2—O8—Tl2C^vii^	72.5 (2)
O6^viii^—Fe1—O9	92.17 (9)	Tl2B—O8—Tl2C^vii^	93.0 (2)
O6—Fe1—O9	87.83 (9)	Tl1B—O8—Tl2C^vii^	141.7 (3)
O4^viii^—Fe1—O9^viii^	92.96 (9)	Tl2A—O8—Tl2C^vii^	97.78 (17)
O4—Fe1—O9^viii^	87.04 (9)	Tl1A—O8—Tl2C^vii^	140.7 (2)
O6^viii^—Fe1—O9^viii^	87.83 (9)	Tl1B^i^—O8—Tl2C^vii^	139.2 (3)
O6—Fe1—O9^viii^	92.17 (9)	Tl2C—O8—Tl2C^vii^	94.4 (4)
O9—Fe1—O9^viii^	180.00 (12)	Tl2B^vii^—O8—Tl2C^vii^	6.5 (2)
O4^viii^—Fe1—Tl2A^vi^	124.04 (9)	As2—O8—Tl2A^vii^	75.34 (10)
O4—Fe1—Tl2A^vi^	55.96 (9)	Tl2B—O8—Tl2A^vii^	90.71 (13)
O6^viii^—Fe1—Tl2A^vi^	33.14 (8)	Tl1B—O8—Tl2A^vii^	140.2 (2)
O6—Fe1—Tl2A^vi^	146.86 (8)	Tl2A—O8—Tl2A^vii^	95.41 (11)
O9—Fe1—Tl2A^vi^	99.80 (8)	Tl1A—O8—Tl2A^vii^	138.90 (9)
O9^viii^—Fe1—Tl2A^vi^	80.20 (8)	Tl1B^i^—O8—Tl2A^vii^	137.1 (2)
O4^viii^—Fe1—Tl2A^xi^	55.96 (9)	Tl2C—O8—Tl2A^vii^	92.0 (2)
O4—Fe1—Tl2A^xi^	124.04 (9)	Tl2B^vii^—O8—Tl2A^vii^	3.75 (10)
O6^viii^—Fe1—Tl2A^xi^	146.86 (8)	Tl2C^vii^—O8—Tl2A^vii^	2.8 (2)
O6—Fe1—Tl2A^xi^	33.14 (8)	As2—O8—Tl2C^xi^	32.11 (14)
O9—Fe1—Tl2A^xi^	80.20 (8)	Tl2B—O8—Tl2C^xi^	115.00 (14)
O9^viii^—Fe1—Tl2A^xi^	99.80 (8)	Tl1B—O8—Tl2C^xi^	152.4 (3)
Tl2A^vi^—Fe1—Tl2A^xi^	180.0	Tl2A—O8—Tl2C^xi^	121.35 (16)
O4^viii^—Fe1—Tl2C^vi^	123.9 (2)	Tl1A—O8—Tl2C^xi^	158.25 (14)
O4—Fe1—Tl2C^vi^	56.1 (2)	Tl1B^i^—O8—Tl2C^xi^	163.7 (3)
O6^viii^—Fe1—Tl2C^vi^	32.4 (2)	Tl2C—O8—Tl2C^xi^	120.0 (4)
O6—Fe1—Tl2C^vi^	147.6 (2)	Tl2B^vii^—O8—Tl2C^xi^	50.72 (11)
O9—Fe1—Tl2C^vi^	96.31 (16)	Tl2C^vii^—O8—Tl2C^xi^	44.3 (3)
O9^viii^—Fe1—Tl2C^vi^	83.69 (16)	Tl2A^vii^—O8—Tl2C^xi^	46.97 (15)
Tl2A^vi^—Fe1—Tl2C^vi^	3.50 (15)	As3—O9—Fe1	126.14 (11)
Tl2A^xi^—Fe1—Tl2C^vi^	176.50 (15)	As3—O9—Tl2B^viii^	100.51 (12)
O4^viii^—Fe1—Tl2C^xi^	56.1 (2)	Fe1—O9—Tl2B^viii^	116.35 (12)
O4—Fe1—Tl2C^xi^	123.9 (2)	As3—O9—Tl2A^viii^	103.06 (11)
O6^viii^—Fe1—Tl2C^xi^	147.6 (2)	Fe1—O9—Tl2A^viii^	115.76 (10)
O6—Fe1—Tl2C^xi^	32.4 (2)	Tl2B^viii^—O9—Tl2A^viii^	3.35 (9)
O9—Fe1—Tl2C^xi^	83.69 (16)	As3—O9—Tl2C^viii^	99.2 (2)
O9^viii^—Fe1—Tl2C^xi^	96.31 (16)	Fe1—O9—Tl2C^viii^	119.46 (19)
Tl2A^vi^—Fe1—Tl2C^xi^	176.50 (15)	Tl2B^viii^—O9—Tl2C^viii^	3.5 (2)
Tl2A^xi^—Fe1—Tl2C^xi^	3.50 (15)	Tl2A^viii^—O9—Tl2C^viii^	4.02 (19)
Tl2C^vi^—Fe1—Tl2C^xi^	180.0	As3—O9—Tl2A^xi^	148.12 (11)
O4^viii^—Fe1—Tl2B^viii^	51.92 (9)	Fe1—O9—Tl2A^xi^	73.23 (8)
O4—Fe1—Tl2B^viii^	128.08 (9)	Tl2B^viii^—O9—Tl2A^xi^	89.41 (8)
O6^viii^—Fe1—Tl2B^viii^	77.24 (7)	Tl2A^viii^—O9—Tl2A^xi^	86.11 (9)
O6—Fe1—Tl2B^viii^	102.76 (7)	Tl2C^viii^—O9—Tl2A^xi^	88.9 (3)
O9—Fe1—Tl2B^viii^	39.51 (8)	As3—O9—Tl2C^xi^	149.74 (18)
O9^viii^—Fe1—Tl2B^viii^	140.49 (8)	Fe1—O9—Tl2C^xi^	70.55 (18)
Tl2A^vi^—Fe1—Tl2B^viii^	103.44 (5)	Tl2B^viii^—O9—Tl2C^xi^	90.8 (2)
Tl2A^xi^—Fe1—Tl2B^viii^	76.56 (5)	Tl2A^viii^—O9—Tl2C^xi^	87.6 (2)
Tl2C^vi^—Fe1—Tl2B^viii^	100.63 (18)	Tl2C^viii^—O9—Tl2C^xi^	90.4 (2)
Tl2C^xi^—Fe1—Tl2B^viii^	79.37 (18)	Tl2A^xi^—O9—Tl2C^xi^	2.7 (2)
O4^viii^—Fe1—Tl2B	128.08 (9)	As3—O9—Tl2B^xi^	148.77 (10)
O4—Fe1—Tl2B	51.92 (9)	Fe1—O9—Tl2B^xi^	75.38 (8)
O6^viii^—Fe1—Tl2B	102.76 (7)	Tl2B^viii^—O9—Tl2B^xi^	85.66 (11)
O6—Fe1—Tl2B	77.24 (7)	Tl2A^viii^—O9—Tl2B^xi^	82.35 (7)
O9—Fe1—Tl2B	140.49 (8)	Tl2C^viii^—O9—Tl2B^xi^	85.1 (2)
O9^viii^—Fe1—Tl2B	39.51 (8)	Tl2A^xi^—O9—Tl2B^xi^	3.77 (8)
Tl2A^vi^—Fe1—Tl2B	76.56 (5)	Tl2C^xi^—O9—Tl2B^xi^	5.8 (2)
Tl2A^xi^—Fe1—Tl2B	103.44 (5)	As3—O9—Tl2C^xiii^	56.57 (16)
Tl2C^vi^—Fe1—Tl2B	79.37 (18)	Fe1—O9—Tl2C^xiii^	95.39 (15)
Tl2C^xi^—Fe1—Tl2B	100.63 (18)	Tl2B^viii^—O9—Tl2C^xiii^	75.0 (2)
Tl2B^viii^—Fe1—Tl2B	180.0	Tl2A^viii^—O9—Tl2C^xiii^	78.28 (18)
O5—Fe2—O11	95.79 (10)	Tl2C^viii^—O9—Tl2C^xiii^	76.7 (4)
O5—Fe2—O1	95.83 (9)	Tl2A^xi^—O9—Tl2C^xiii^	154.43 (14)
O11—Fe2—O1	94.90 (9)	Tl2C^xi^—O9—Tl2C^xiii^	153.4 (3)
O5—Fe2—O10^x^	87.53 (9)	Tl2B^xi^—O9—Tl2C^xiii^	152.26 (17)
O11—Fe2—O10^x^	91.37 (9)	As3—O9—Tl2C^vi^	71.33 (15)
O1—Fe2—O10^x^	172.54 (9)	Fe1—O9—Tl2C^vi^	60.13 (14)
O5—Fe2—O7^ii^	94.10 (9)	Tl2B^viii^—O9—Tl2C^vi^	110.09 (17)
O11—Fe2—O7^ii^	169.50 (9)	Tl2A^viii^—O9—Tl2C^vi^	112.86 (18)
O1—Fe2—O7^ii^	87.58 (9)	Tl2C^viii^—O9—Tl2C^vi^	112.9 (3)
O10^x^—Fe2—O7^ii^	85.53 (9)	Tl2A^xi^—O9—Tl2C^vi^	133.37 (12)
O5—Fe2—O3^iv^	174.72 (8)	Tl2C^xi^—O9—Tl2C^vi^	130.68 (17)
O11—Fe2—O3^iv^	85.61 (9)	Tl2B^xi^—O9—Tl2C^vi^	135.38 (17)
O1—Fe2—O3^iv^	89.11 (8)	Tl2C^xiii^—O9—Tl2C^vi^	41.7 (3)
O10^x^—Fe2—O3^iv^	87.34 (8)	As3—O9—Tl2B^xiii^	56.73 (8)
O7^ii^—Fe2—O3^iv^	84.23 (9)	Fe1—O9—Tl2B^xiii^	99.49 (9)
O5—Fe2—Tl1B	83.71 (16)	Tl2B^viii^—O9—Tl2B^xiii^	70.07 (7)
O11—Fe2—Tl1B	134.23 (14)	Tl2A^viii^—O9—Tl2B^xiii^	73.39 (8)
O1—Fe2—Tl1B	40.21 (13)	Tl2C^viii^—O9—Tl2B^xiii^	71.7 (2)
O10^x^—Fe2—Tl1B	134.14 (13)	Tl2A^xi^—O9—Tl2B^xiii^	152.83 (7)
O7^ii^—Fe2—Tl1B	50.57 (15)	Tl2C^xi^—O9—Tl2B^xiii^	152.35 (17)
O3^iv^—Fe2—Tl1B	98.97 (16)	Tl2B^xi^—O9—Tl2B^xiii^	150.11 (7)
O5—Fe2—Tl2C^xii^	135.2 (2)	Tl2C^xiii^—O9—Tl2B^xiii^	5.39 (19)
O11—Fe2—Tl2C^xii^	115.68 (19)	Tl2C^vi^—O9—Tl2B^xiii^	46.99 (13)
O1—Fe2—Tl2C^xii^	111.3 (3)	As3—O10—Fe2^x^	135.25 (12)
O10^x^—Fe2—Tl2C^xii^	62.1 (3)	As3—O10—Tl2B^viii^	98.29 (12)
O7^ii^—Fe2—Tl2C^xii^	54.13 (19)	Fe2^x^—O10—Tl2B^viii^	93.44 (9)
O3^iv^—Fe2—Tl2C^xii^	40.4 (2)	As3—O10—Tl2C^viii^	105.5 (2)
Tl1B—Fe2—Tl2C^xii^	94.5 (3)	Fe2^x^—O10—Tl2C^viii^	87.1 (2)
O5—Fe2—Tl2B^xii^	133.55 (8)	Tl2B^viii^—O10—Tl2C^viii^	7.4 (3)
O11—Fe2—Tl2B^xii^	111.03 (9)	As3—O10—Tl2A^viii^	101.32 (11)
O1—Fe2—Tl2B^xii^	117.77 (7)	Fe2^x^—O10—Tl2A^viii^	89.85 (10)
O10^x^—Fe2—Tl2B^xii^	55.86 (7)	Tl2B^viii^—O10—Tl2A^viii^	3.64 (12)
O7^ii^—Fe2—Tl2B^xii^	59.09 (9)	Tl2C^viii^—O10—Tl2A^viii^	4.2 (2)
O3^iv^—Fe2—Tl2B^xii^	41.56 (7)	As3—O10—Tl1B^xiii^	95.8 (2)
Tl1B—Fe2—Tl2B^xii^	101.28 (17)	Fe2^x^—O10—Tl1B^xiii^	101.6 (2)
Tl2C^xii^—Fe2—Tl2B^xii^	7.1 (2)	Tl2B^viii^—O10—Tl1B^xiii^	140.98 (15)
O5—Fe2—Tl2A^xii^	136.51 (9)	Tl2C^viii^—O10—Tl1B^xiii^	139.3 (3)
O11—Fe2—Tl2A^xii^	112.58 (9)	Tl2A^viii^—O10—Tl1B^xiii^	141.70 (15)
O1—Fe2—Tl2A^xii^	112.92 (9)	As3—O10—Tl1A^xiii^	91.72 (8)
O10^x^—Fe2—Tl2A^xii^	60.69 (9)	Fe2^x^—O10—Tl1A^xiii^	106.28 (8)
O7^ii^—Fe2—Tl2A^xii^	57.26 (8)	Tl2B^viii^—O10—Tl1A^xiii^	139.97 (8)
O3^iv^—Fe2—Tl2A^xii^	38.83 (8)	Tl2C^viii^—O10—Tl1A^xiii^	139.3 (2)
Tl1B—Fe2—Tl2A^xii^	97.41 (17)	Tl2A^viii^—O10—Tl1A^xiii^	141.15 (8)
Tl2C^xii^—Fe2—Tl2A^xii^	3.2 (2)	Tl1B^xiii^—O10—Tl1A^xiii^	4.7 (2)
Tl2B^xii^—Fe2—Tl2A^xii^	4.85 (8)	As3—O10—Tl1B^iv^	87.8 (2)
O5—Fe2—Tl1A	84.15 (7)	Fe2^x^—O10—Tl1B^iv^	110.8 (2)
O11—Fe2—Tl1A	131.70 (7)	Tl2B^viii^—O10—Tl1B^iv^	138.59 (14)
O1—Fe2—Tl1A	37.68 (6)	Tl2C^viii^—O10—Tl1B^iv^	138.8 (3)
O10^x^—Fe2—Tl1A	136.69 (6)	Tl2A^viii^—O10—Tl1B^iv^	140.17 (14)
O7^ii^—Fe2—Tl1A	52.97 (6)	Tl1B^xiii^—O10—Tl1B^iv^	9.3 (4)
O3^iv^—Fe2—Tl1A	98.74 (6)	Tl1A^xiii^—O10—Tl1B^iv^	4.6 (2)
Tl1B—Fe2—Tl1A	2.55 (12)	As3—O10—Tl2B^xiii^	84.04 (10)
Tl2C^xii^—Fe2—Tl1A	95.9 (2)	Fe2^x^—O10—Tl2B^xiii^	140.47 (10)
Tl2B^xii^—Fe2—Tl1A	102.81 (6)	Tl2B^viii^—O10—Tl2B^xiii^	82.48 (8)
Tl2A^xii^—Fe2—Tl1A	98.84 (7)	Tl2C^viii^—O10—Tl2B^xiii^	84.9 (2)
O5—Fe2—Tl1B^i^	84.53 (14)	Tl2A^viii^—O10—Tl2B^xiii^	84.87 (9)
O11—Fe2—Tl1B^i^	129.48 (12)	Tl1B^xiii^—O10—Tl2B^xiii^	63.04 (16)
O1—Fe2—Tl1B^i^	35.47 (11)	Tl1A^xiii^—O10—Tl2B^xiii^	60.05 (6)
O10^x^—Fe2—Tl1B^i^	138.92 (11)	Tl1B^iv^—O10—Tl2B^xiii^	57.31 (15)
O7^ii^—Fe2—Tl1B^i^	55.08 (13)	As3—O10—Tl2A^xiii^	81.21 (9)
O3^iv^—Fe2—Tl1B^i^	98.53 (14)	Fe2^x^—O10—Tl2A^xiii^	142.78 (10)
Tl1B—Fe2—Tl1B^i^	4.8 (2)	Tl2B^viii^—O10—Tl2A^xiii^	86.56 (9)
Tl2C^xii^—Fe2—Tl1B^i^	97.2 (3)	Tl2C^viii^—O10—Tl2A^xiii^	89.2 (2)
Tl2B^xii^—Fe2—Tl1B^i^	104.14 (15)	Tl2A^viii^—O10—Tl2A^xiii^	89.05 (10)
Tl2A^xii^—Fe2—Tl1B^i^	100.08 (15)	Tl1B^xiii^—O10—Tl2A^xiii^	59.95 (17)
Tl1A—Fe2—Tl1B^i^	2.23 (10)	Tl1A^xiii^—O10—Tl2A^xiii^	56.72 (6)
O4—As1—O1	111.35 (11)	Tl1B^iv^—O10—Tl2A^xiii^	53.75 (16)
O4—As1—O3	108.09 (11)	Tl2B^xiii^—O10—Tl2A^xiii^	4.64 (6)
O1—As1—O3	117.28 (10)	As3—O10—Tl2C^xiii^	78.23 (19)
O4—As1—O2	105.89 (12)	Fe2^x^—O10—Tl2C^xiii^	146.03 (18)
O1—As1—O2	103.87 (11)	Tl2B^viii^—O10—Tl2C^xiii^	85.1 (2)
O3—As1—O2	109.74 (11)	Tl2C^viii^—O10—Tl2C^xiii^	88.2 (4)
O4—As1—Tl2B^vi^	82.50 (10)	Tl2A^viii^—O10—Tl2C^xiii^	87.7 (2)
O1—As1—Tl2B^vi^	161.67 (10)	Tl1B^xiii^—O10—Tl2C^xiii^	62.5 (2)
O3—As1—Tl2B^vi^	44.89 (11)	Tl1A^xiii^—O10—Tl2C^xiii^	59.12 (19)
O2—As1—Tl2B^vi^	82.71 (11)	Tl1B^iv^—O10—Tl2C^xiii^	55.9 (2)
O4—As1—Tl2A^vi^	77.17 (11)	Tl2B^xiii^—O10—Tl2C^xiii^	6.0 (2)
O1—As1—Tl2A^vi^	163.46 (9)	Tl2A^xiii^—O10—Tl2C^xiii^	3.57 (13)
O3—As1—Tl2A^vi^	46.32 (9)	As3—O11—Fe2	147.97 (13)
O2—As1—Tl2A^vi^	86.55 (11)	As3—O11—Tl1B^iv^	74.11 (15)
Tl2B^vi^—As1—Tl2A^vi^	5.82 (8)	Fe2—O11—Tl1B^iv^	136.20 (15)
O4—As1—Tl1B	109.71 (17)	As3—O11—Tl1A^xiii^	74.20 (8)
O1—As1—Tl1B	41.4 (2)	Fe2—O11—Tl1A^xiii^	136.91 (9)
O3—As1—Tl1B	141.82 (17)	Tl1B^iv^—O11—Tl1A^xiii^	4.21 (18)
O2—As1—Tl1B	64.3 (2)	As3—O11—Tl1B^xiii^	74.37 (14)
Tl2B^vi^—As1—Tl1B	146.8 (2)	Fe2—O11—Tl1B^xiii^	137.28 (15)
Tl2A^vi^—As1—Tl1B	150.9 (2)	Tl1B^iv^—O11—Tl1B^xiii^	8.3 (4)
O4—As1—Tl2C^vi^	78.6 (3)	Tl1A^xiii^—O11—Tl1B^xiii^	4.13 (17)
O1—As1—Tl2C^vi^	159.27 (18)	As3—O11—Tl2B^xii^	159.69 (11)
O3—As1—Tl2C^vi^	42.3 (2)	Fe2—O11—Tl2B^xii^	47.38 (8)
O2—As1—Tl2C^vi^	90.0 (2)	Tl1B^iv^—O11—Tl2B^xii^	97.08 (15)
Tl2B^vi^—As1—Tl2C^vi^	7.5 (3)	Tl1A^xiii^—O11—Tl2B^xii^	95.68 (7)
Tl2A^vi^—As1—Tl2C^vi^	4.29 (16)	Tl1B^xiii^—O11—Tl2B^xii^	94.27 (15)
Tl1B—As1—Tl2C^vi^	154.2 (3)	As3—O11—Tl2C^xii^	164.81 (18)
O4—As1—Tl1A	110.64 (9)	Fe2—O11—Tl2C^xii^	43.96 (14)
O1—As1—Tl1A	46.10 (7)	Tl1B^iv^—O11—Tl2C^xii^	98.2 (2)
O3—As1—Tl1A	141.27 (7)	Tl1A^xiii^—O11—Tl2C^xii^	97.17 (15)
O2—As1—Tl1A	59.36 (9)	Tl1B^xiii^—O11—Tl2C^xii^	96.12 (19)
Tl2B^vi^—As1—Tl1A	141.78 (6)	Tl2B^xii^—O11—Tl2C^xii^	5.36 (18)
Tl2A^vi^—As1—Tl1A	145.90 (8)	As3—O12—Tl2C^xiii^	142.6 (4)
Tl1B—As1—Tl1A	5.0 (2)	As3—O12—Tl2A^xiii^	140.85 (15)
Tl2C^vi^—As1—Tl1A	149.2 (2)	Tl2C^xiii^—O12—Tl2A^xiii^	5.1 (2)
O4—As1—Tl1B^i^	111.38 (17)	As3—O12—Tl2B^xiii^	134.29 (12)
O1—As1—Tl1B^i^	50.7 (2)	Tl2C^xiii^—O12—Tl2B^xiii^	8.8 (3)
O3—As1—Tl1B^i^	140.23 (15)	Tl2A^xiii^—O12—Tl2B^xiii^	6.95 (9)
O2—As1—Tl1B^i^	54.5 (2)	As3—O12—Tl2C^vi^	136.8 (2)
Tl2B^vi^—As1—Tl1B^i^	137.0 (2)	Tl2C^xiii^—O12—Tl2C^vi^	69.0 (4)
Tl2A^vi^—As1—Tl1B^i^	141.1 (2)	Tl2A^xiii^—O12—Tl2C^vi^	73.2 (2)
Tl1B—As1—Tl1B^i^	9.8 (4)	Tl2B^xiii^—O12—Tl2C^vi^	77.4 (2)
Tl2C^vi^—As1—Tl1B^i^	144.4 (3)	As3—O12—Tl1B^iv^	105.94 (19)
Tl1A—As1—Tl1B^i^	4.8 (2)	Tl2C^xiii^—O12—Tl1B^iv^	76.4 (3)
O4—As1—Tl2B	65.74 (9)	Tl2A^xiii^—O12—Tl1B^iv^	71.88 (15)
O1—As1—Tl2B	104.61 (8)	Tl2B^xiii^—O12—Tl1B^iv^	74.80 (17)
O3—As1—Tl2B	135.82 (8)	Tl2C^vi^—O12—Tl1B^iv^	111.5 (3)
O2—As1—Tl2B	42.84 (9)	As3—O12—Tl1A^xiii^	101.85 (10)
Tl2B^vi^—As1—Tl2B	91.89 (9)	Tl2C^xiii^—O12—Tl1A^xiii^	78.1 (2)
Tl2A^vi^—As1—Tl2B	91.78 (7)	Tl2A^xiii^—O12—Tl1A^xiii^	73.36 (9)
Tl1B—As1—Tl2B	67.5 (2)	Tl2B^xiii^—O12—Tl1A^xiii^	75.74 (10)
Tl2C^vi^—As1—Tl2B	96.05 (17)	Tl2C^vi^—O12—Tl1A^xiii^	116.3 (2)
Tl1A—As1—Tl2B	64.33 (4)	Tl1B^iv^—O12—Tl1A^xiii^	4.8 (2)
Tl1B^i^—As1—Tl2B	61.4 (2)	As3—O12—Tl2A^vi^	133.49 (12)
O4—As1—Tl2A	70.99 (11)	Tl2C^xiii^—O12—Tl2A^vi^	71.4 (3)
O1—As1—Tl2A	103.97 (9)	Tl2A^xiii^—O12—Tl2A^vi^	75.76 (13)
O3—As1—Tl2A	134.43 (9)	Tl2B^xiii^—O12—Tl2A^vi^	79.68 (10)
O2—As1—Tl2A	37.60 (11)	Tl2C^vi^—O12—Tl2A^vi^	3.4 (2)
Tl2B^vi^—As1—Tl2A	91.71 (10)	Tl1B^iv^—O12—Tl2A^vi^	114.7 (2)
Tl2A^vi^—As1—Tl2A	92.14 (8)	Tl1A^xiii^—O12—Tl2A^vi^	119.42 (8)
Tl1B—As1—Tl2A	65.3 (2)	As3—O12—Tl1B^xiii^	98.06 (16)
Tl2C^vi^—As1—Tl2A	96.43 (18)	Tl2C^xiii^—O12—Tl1B^xiii^	79.6 (3)
Tl1A—As1—Tl2A	61.81 (6)	Tl2A^xiii^—O12—Tl1B^xiii^	74.82 (14)
Tl1B^i^—As1—Tl2A	58.5 (2)	Tl2B^xiii^—O12—Tl1B^xiii^	76.69 (16)
Tl2B—As1—Tl2A	5.35 (7)	Tl2C^vi^—O12—Tl1B^xiii^	120.6 (3)
O6—As2—O5	112.19 (11)	Tl1B^iv^—O12—Tl1B^xiii^	9.2 (4)
O6—As2—O7	108.23 (11)	Tl1A^xiii^—O12—Tl1B^xiii^	4.42 (17)
O5—As2—O7	109.50 (11)	Tl2A^vi^—O12—Tl1B^xiii^	123.78 (19)
O6—As2—O8	108.63 (11)	As3—O12—Tl2B^vi^	134.55 (12)
O5—As2—O8	111.53 (11)	Tl2C^xiii^—O12—Tl2B^vi^	73.0 (3)
O7—As2—O8	106.56 (10)	Tl2A^xiii^—O12—Tl2B^vi^	77.09 (9)
O6—As2—Tl1B^ii^	102.43 (17)	Tl2B^xiii^—O12—Tl2B^vi^	81.46 (12)
O5—As2—Tl1B^ii^	62.73 (19)	Tl2C^vi^—O12—Tl2B^vi^	4.5 (2)
O7—As2—Tl1B^ii^	53.70 (19)	Tl1B^iv^—O12—Tl2B^vi^	111.1 (2)
O8—As2—Tl1B^ii^	147.66 (17)	Tl1A^xiii^—O12—Tl2B^vi^	115.93 (9)
O6—As2—Tl2A^xi^	48.51 (9)	Tl2A^vi^—O12—Tl2B^vi^	4.80 (8)
O5—As2—Tl2A^xi^	110.40 (10)	Tl1B^xiii^—O12—Tl2B^vi^	120.33 (19)
O7—As2—Tl2A^xi^	63.33 (9)	As3—O12—Tl2B^viii^	57.27 (8)
O8—As2—Tl2A^xi^	137.72 (10)	Tl2C^xiii^—O12—Tl2B^viii^	87.6 (4)
Tl1B^ii^—As2—Tl2A^xi^	61.81 (17)	Tl2A^xiii^—O12—Tl2B^viii^	87.72 (12)
O6—As2—Tl2C^xi^	48.7 (2)	Tl2B^xiii^—O12—Tl2B^viii^	80.79 (8)
O5—As2—Tl2C^xi^	114.46 (19)	Tl2C^vi^—O12—Tl2B^viii^	119.4 (3)
O7—As2—Tl2C^xi^	61.6 (2)	Tl1B^iv^—O12—Tl2B^viii^	115.9 (2)
O8—As2—Tl2C^xi^	133.80 (19)	Tl1A^xiii^—O12—Tl2B^viii^	111.69 (9)
Tl1B^ii^—As2—Tl2C^xi^	64.3 (2)	Tl2A^vi^—O12—Tl2B^viii^	117.69 (10)
Tl2A^xi^—As2—Tl2C^xi^	4.18 (17)	Tl1B^xiii^—O12—Tl2B^viii^	107.8 (2)
O6—As2—Tl1A^xi^	101.68 (8)	Tl2B^vi^—O12—Tl2B^viii^	122.47 (11)
O5—As2—Tl1A^xi^	61.84 (8)	As3—O12—Tl2A^viii^	57.41 (8)
O7—As2—Tl1A^xi^	55.05 (7)	Tl2C^xiii^—O12—Tl2A^viii^	87.4 (4)
O8—As2—Tl1A^xi^	148.71 (8)	Tl2A^xiii^—O12—Tl2A^viii^	87.47 (11)
Tl1B^ii^—As2—Tl1A^xi^	1.42 (17)	Tl2B^xiii^—O12—Tl2A^viii^	80.53 (8)
Tl2A^xi^—As2—Tl1A^xi^	61.71 (6)	Tl2C^vi^—O12—Tl2A^viii^	119.6 (3)
Tl2C^xi^—As2—Tl1A^xi^	64.28 (19)	Tl1B^iv^—O12—Tl2A^viii^	115.5 (2)
O6—As2—Tl2C^vii^	77.7 (2)	Tl1A^xiii^—O12—Tl2A^viii^	111.30 (7)
O5—As2—Tl2C^vii^	156.3 (2)	Tl2A^vi^—O12—Tl2A^viii^	117.93 (10)
O7—As2—Tl2C^vii^	47.2 (2)	Tl1B^xiii^—O12—Tl2A^viii^	107.4 (2)
O8—As2—Tl2C^vii^	83.6 (3)	Tl2B^vi^—O12—Tl2A^viii^	122.72 (7)
Tl1B^ii^—As2—Tl2C^vii^	94.6 (3)	Tl2B^viii^—O12—Tl2A^viii^	0.42 (11)
Tl2A^xi^—As2—Tl2C^vii^	59.0 (2)	As3—O12—H12	101 (3)
Tl2C^xi^—As2—Tl2C^vii^	54.9 (4)	Tl2C^xiii^—O12—H12	116 (3)
Tl1A^xi^—As2—Tl2C^vii^	95.6 (2)	Tl2A^xiii^—O12—H12	118 (3)
O6—As2—Tl2B^xi^	48.84 (8)	Tl2B^xiii^—O12—H12	125 (3)
O5—As2—Tl2B^xi^	109.53 (11)	Tl2C^vi^—O12—H12	56 (3)
O7—As2—Tl2B^xi^	63.38 (9)	Tl1B^iv^—O12—H12	95 (3)
O8—As2—Tl2B^xi^	138.60 (11)	Tl1A^xiii^—O12—H12	98 (3)
Tl1B^ii^—As2—Tl2B^xi^	61.06 (16)	Tl2A^vi^—O12—H12	56 (3)
Tl2A^xi^—As2—Tl2B^xi^	0.88 (12)	Tl1B^xiii^—O12—H12	101 (3)
Tl2C^xi^—As2—Tl2B^xi^	5.0 (2)	Tl2B^vi^—O12—H12	52 (3)
Tl1A^xi^—As2—Tl2B^xi^	60.95 (5)	Tl2B^viii^—O12—H12	146 (3)
Tl2C^vii^—As2—Tl2B^xi^	59.7 (2)	Tl2A^viii^—O12—H12	146 (3)
O6—As2—Tl1B	136.29 (14)		

Symmetry codes: (i) −*x*+1, −*y*+2, −*z*; (ii) −*x*, −*y*+2, −*z*; (iii) *x*+1, *y*, *z*; (iv) −*x*+1, −*y*+1, −*z*; (v) *x*, *y*+1, *z*; (vi) −*x*+1, −*y*+1, −*z*+1; (vii) −*x*, −*y*+2, −*z*+1; (viii) −*x*, −*y*+1, −*z*+1; (ix) −*x*+1, −*y*+2, −*z*+1; (x) −*x*, −*y*+1, −*z*; (xi) *x*−1, *y*, *z*; (xii) *x*, *y*, *z*−1; (xiii) *x*, *y*−1, *z*.

### Thallium iron bis[hydrogen arsenate(V)] (TlFeHAsO42) Hydrogen-bond geometry (Å, º)

**Table:**

*D*—H···*A*	*D*—H	H···*A*	*D*···*A*	*D*—H···*A*
O2—H2···O9^iii^	0.85 (3)	1.86 (3)	2.707 (3)	176 (5)
O8—H8···O10^v^	0.982 (2)	1.598 (2)	2.569 (3)	169.44 (15)
O12—H12···O3	0.88 (3)	1.86 (3)	2.729 (3)	172 (5)

Symmetry codes: (iii) *x*+1, *y*, *z*; (v) *x*, *y*+1, *z*.
